# Genes in the Ureteric Budding Pathway: Association Study on Vesico-Ureteral Reflux Patients

**DOI:** 10.1371/journal.pone.0031327

**Published:** 2012-04-27

**Authors:** Albertien M. van Eerde, Karen Duran, Els van Riel, Carolien G. F. de Kovel, Bobby P. C. Koeleman, Nine V. A. M. Knoers, Kirsten Y. Renkema, Henricus J. R. van der Horst, Arend Bökenkamp, Johanna M. van Hagen, Leonard H. van den Berg, Katja P. Wolffenbuttel, Joop van den Hoek, Wouter F. Feitz, Tom P. V. M. de Jong, Jacques C. Giltay, Cisca Wijmenga

**Affiliations:** 1 Department of Medical Genetics, University Medical Center Utrecht, Utrecht, The Netherlands; 2 Department of Pediatric Urology, VU University Medical Center, Amsterdam, The Netherlands; 3 Department of Pediatric Nephrology, VU University Medical Center, Amsterdam, The Netherlands; 4 Department of Human Genetics, VU University Medical Center, Amsterdam, The Netherlands; 5 Department of Neurology, Rudolf Magnus Institute of Neuroscience, University Medical Center Utrecht, Utrecht, The Netherlands; 6 Department of Pediatric Urology, Sophia Children’s Hospital, Erasmus Medical Center, Rotterdam, The Netherlands; 7 Department of Urology, Pediatric Urology Centre, Radboud University Nijmegen Medical Centre, Nijmegen, The Netherlands; 8 Pediatric Renal Center, University Medical Center Utrecht and Academic Medical Center. Amsterdam, The Netherlands; 9 Department of Genetics, University Medical Center Groningen and University of Groningen, The Netherlands; Ohio State University Medical Center, United States of America

## Abstract

Vesico-ureteral reflux (VUR) is the retrograde passage of urine from the bladder to the urinary tract and causes 8.5% of end-stage renal disease in children. It is a complex genetic developmental disorder, in which ectopic embryonal ureteric budding is implicated in the pathogenesis. VUR is part of the spectrum of Congenital Anomalies of the Kidney and Urinary Tract (CAKUT). We performed an extensive association study for primary VUR using a two-stage, case-control design, investigating 44 candidate genes in the ureteric budding pathway in 409 Dutch VUR patients. The 44 genes were selected from the literature and a set of 567 single nucleotide polymorphisms (SNPs) capturing their genetic variation was genotyped in 207 cases and 554 controls. The 14 SNPs with p<0.005 were included in a follow-up study in 202 cases and 892 controls. Of the total cohort, ∼50% showed a clear-cut primary VUR phenotype and ∼25% had both a duplex collecting system and VUR. We also looked for association in these two extreme phenotype groups. None of the SNPs reached a significant p-value. Common genetic variants in four genes (*GREM1*, *EYA1*, *ROBO2* and *UPK3A*) show a trend towards association with the development of primary VUR (*GREM1*, *EYA1*, *ROBO2*) or duplex collecting system (*EYA1* and *UPK3A*). SNPs in three genes (*TGFB1*, *GNB3* and *VEGFA*) have been shown to be associated with VUR in other populations. Only the result of rs1800469 in *TGFB1* hinted at association in our study. This is the first extensive study of common variants in the genes of the ureteric budding pathway and the genetic susceptibility to primary VUR.

## Introduction

Vesico-ureteral reflux [VUR (MIM 193000)] is the retrograde passage of urine from the bladder into the upper urinary tract. It is one of the most commonly detected congenital anomalies and probably has a conservatively estimated prevalence of 1%. [1], [2] It has a primary and a secondary form: primary VUR is due to an incompetent valve mechanism at the uretero-vesical junction, while secondary VUR is due to a functional or anatomical urethral obstruction. VUR is a developmental disorder, which may occur in isolation or as part of a Mendelian or other syndrome. The Winter-Baraitser Dysmorphology Database lists 68 syndromes with ‘urinary reflux’. [3]

Although most children grow out of the disorder without serious morbidity, a subset does develop long-term complications. In this group VUR results in renal damage, either as a result of ascending urinary tract infections (reflux nephropathy) or of renal hypo- or dysplasia, which is often associated with VUR. As such, in these two groups VUR accounts for 7.4 – 9.6% and 8.8 – 13.8%, respectively, of end-stage renal disease in Dutch children. [4]

Clinical observations and the results of many studies support the notion that there is a heterogeneous genetic basis for VUR. The incidence of VUR is increased in first-degree relatives of patients [5]–[7] and there is 80% concordance between monozygotic twins. [8] In a subset of families, the segregation pattern suggests autosomal dominant inheritance with variable penetrance. [9]–[11] Other inheritance patterns, including polygenic, have also been observed. [12]–[14] Linkage studies have revealed different loci linked to VUR, although most loci have not been convincingly replicated. [10], [11], [15]–[19] Work in knock-out mice has confirmed the importance of genetic factors in the etiology of VUR. [20] Evidence for a continuous distribution of anatomic parameters, like the length of the intravesical ureter and the position of ureteric budding from the mesonephric duct, associated with VUR suggests that these parameters are quantitative traits encoded by multiple genes. [20] In common complex diseases, common genetic variants are thought to be part of the genetic disease component. [21], [22] Because of their modest individual effect size, common variants are not detected by a linkage approach.

To date, no major susceptibility genes have been identified for VUR. [10],[11],[15]–[17],[19],[20],[23] However, since embryonal ectopic ureteric budding has been proposed as a mechanism for the development of VUR, [24], [25] genes involved in this process are considered to be potential candidate genes for VUR susceptibility (see Schedl [60]: figure 3: http://www.nature.com/nrg/journal/v8/n10/fig_tab/nrg2205_F3.html#figure-title). In particular, ectopic or deficient ureteric budding can lead to a diverse spectrum of phenotypes known as “congenital anomalies of the kidney and urinary tract” or CAKUT. CAKUT include VUR, hypo-/dysplastic kidneys and duplex collecting systems. Variable combinations of these phenotypes are seen in sibships (both in mice and humans) suggesting that the same genetic variation is causally involved in the whole CAKUT spectrum. [25] For example, defects of the *RET* (ENSG00000165731) and *GDNF* (ENSG00000168621) genes have been shown to cause deficient ureteric budding with malformed or absent kidneys. [26], [27] *ROBO2* (ENSG00000185008) regulates the expression of *GDNF* [28] and was shown to be mutated in a small number of VUR/CAKUT patients. [29] Genes involved in the *RET/GDNF* pathway are obvious functional candidate genes for VUR. Genes involved in syndromal VUR, like *EYA1* in Branchiootorenal Syndrome (MIM 113650) and *PAX2* in Papillorenal Syndrome (MIM 167409), are often also implicated in the ureteric budding pathway and thus attractive candidate genes as well. Hence, we hypothesize that common variants in genes in the ureteric budding pathway contribute to the genetic susceptibility for primary VUR.

We describe the first genetic association study in VUR patients, targeted to a large set of candidate genes primarily involved in the ureteric budding pathway.

**Figure 1:**
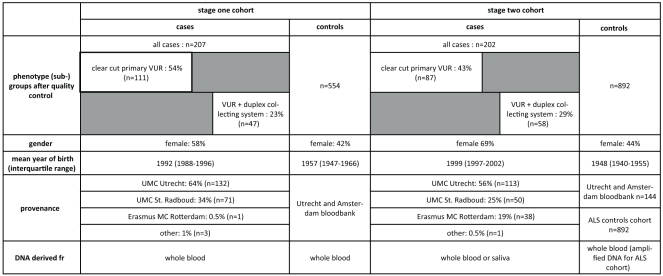
Case-control cohorts. Detailed overview of the two Dutch case-control cohorts and two phenotype subgroups in which the association study was performed.

## Results

We used a two-stage approach in which all of the designed SNPs were genotyped in the first stage. Then a number of top SNPs were chosen to be genotyped in the second stage. The joint analysis of both stages is the end result of the study. [30]

In stage one, we successfully genotyped 567 (out of 758) SNPs (single nucleotide polymorphisms) in 44 genes (Tables S1, S2 and S4) for association analysis in a cohort of 207 primary VUR patients and 554 controls (Figure 1). Examples of these 44 genes are: *BMP4, EYA1, FOXC1, GDNF, RET, GFRA1, ITGA8, PAX2, SALL1, ROBO2* and *SLIT2*. We also performed a subset analysis in two extreme phenotype subgroups: (1) a group of 111 clear-cut primary VUR cases (e.g. patients with mild dysfunctional voiding, a minor relative meatal stenosis, or insignificant urethral valves were excluded) and (2) a group of 47 patients with VUR and a uni- or bilateral, complete or incomplete, duplex collecting system.

The stage one p-values of the overall and two subset analyses were combined in one list and ranked according to p-value (data not shown). We set out to genotype the top 14 SNPs, which mapped to *RARB, ROBO2*, *EYA1, GFRA1*, *GREM1* and *UPK3A*, in stage two (Table 1). By choosing to genotype the top 14 SNPs the p-value threshold for the first stage was set to 0.005 (data not shown). The stage two cohort of 202 cases and 892 controls was also subjected to the subset analyses (87 clear-cut primary VUR cases, and 58 cases with a duplex collecting system and VUR) (Figure 1).

**Table 1:** Results of the joint, stage one and stage two analyses of the 14 SNPs tested for association in stage two.

SNP	gene	chromosome	basepair position	minor allele (major allele)	MAF (controls; joint unless not genotyped in stage two)	MAF (all cases; joint unless not genotyped in stage 2)	stage 1 p-value all cases *	stage 2 p-value all cases *	joint p-value all cases **	OR all cases (95% CI)	MAF (clear cut primary VUR cases; joint unless not genotyped in stage 2)	stage 1 p-value clear cut primary VUR cases *	stage 2 p-value clear cut primary VUR cases *	joint p-value clear cut primary VUR cases **	OR clear cut primary VUR cases (95% CI)	MAF (case duplex collecting system; joint unless not genotyped in stage 2)	stage 1 p-value duplex collecting system+VUR cases *	stage 2 p-value duplex collecting system+VUR cases *	joint p-value duplex collecting system+VUR cases **	OR duplex collecting system+VUR cases (95% CI)
rs6780105	*RARB*	3	25278709	G (C)	0.18	0.15	0.0024#	0.765	0.019	0.77 (0.62 – 0.96)	0.15	0.0435	0.979	0.134	0.80 (0.60 – 1.07)	0.11	0.0155	0.237	0.012	0.57 (0.37 – 0.89)
rs755661	*RARB*	3	25447044	A (G)	0.45	0.46	0.3350	0.179	0.763	1.02 (0.88 – 1.20)	0.47	0.7664	0.424	0.448	1.09 (0.88 – 1.34)	0.42	0.0052#	0.223	0.344	0.87 (0.66 – 1.16)
rs4476545$	*ROBO2*	3	77193190	C (G)	0.15	0.17	0.0402	0.879	0.136	1.18 (0.95 – 1.46)	0.20	0.0020#	0.381	0.005	1.48 (1.13 – 1.94)	0.13	0.9889	0.574	0.658	0.91 (0.60 – 1.38)
rs1666130$	*ROBO2*	3	77681633	G (A)	0.49	0.44	0.0042#	0.321	0.007	0.80 (0.69 – 0.94)	0.43	0.0257	0.201	0.012	0.76 (0.61 – 0.94)	0.49	0.8533	0.874	0.996	1.00 (0.75 – 1.33)
rs1403848***	*ROBO2*	3	77692345	C (A)	0.50	0.41	0.0025#	***	***	***	0.41	0.0199	***	***	***	0.48	0.6920	***	***	***
rs10103397****	*EYA1*	8	72274153	G (A)	0.26	0.32	0.0075	****	****	****	0.36	0.0020#	****	****	****	0.31	0.2554	****	****	****
rs9298164****	*EYA1*	8	72289193	A (G)	0.24	0.32	0.0047#	****	****	****	0.34	0.0025#	****	****	****	0.31	0.1691	****	****	****
rs3735935$	*EYA1*	8	72290318	A (C)	0.25	0.30	0.0046#	0.306	0.007	1.27 (1.07 – 1.51)	0.30	0.0023#	0.752	0.043	1.27 (1.01 – 1.61)	0.31	0.2107	0.116	0.045	1.36 (1.01 – 1.85)
rs1481800	*EYA1*	8	72293980	A (G)	0.23	0.27	0.0128	0.746	0.051	1.20 (1.00 – 1.43)	0.26	0.0052#	0.217	0.224	1.16 (0.91 – 1.49)	0.29	0.4201	0.100	0.076	1.33 (0.97 – 1.81)
rs11197571	*GFRA1*	10	117932624	G (A)	0.13	0.16	0.0143	0.468	0.026	1.28 (1.03 – 1.59)	0.16	0.0542	0.975	0.163	1.23 (0.92 – 1.66)	0.18	0.0037#	0.909	0.063	1.42 (0.98 – 2.06)
rs7497354$	*GREM1*	15	30802694	G (A)	0.39	0.33	0.0030#	0.148	0.002	0.77 (0.65 – 0.91)	0.33	0.1936	0.055	0.024	0.77 (0.62 – 0.97)	0.33	0.0798	0.501	0.096	0.78 (0.58 – 1.05)
rs1057353$	*UPK3A*	22	44061968	G (C)	0.22	0.24	0.6806	0.293	0.294	1.10 (0.92 – 1.33)	0.21	0.2785	0.680	0.619	0.94 (0.72 – 1.21)	0.30	0.0020#	0.421	0.008	1.52 (1.11 – 2.06)
rs1135360	*UPK3A*	22	44063666	G (A)	0.41	0.45	0.2655	0.175	0.080	1.15 (0.98 – 1.35)	0.43	0.6395	0.141	0.509	1.08 (0.87 – 1.33)	0.47	0.0023#	0.588	0.103	1.26 (0.95 – 1.67)
rs3788643	*UPK3A*	22	44064754	A (G)	0.15	0.16	0.7451	0.291	0.320	1.12 (0.90 – 1.38)	0.15	0.1938	0.064	0.718	1.06 (0.79 – 1.42)	0.20	0.0011#	0.838	0.020	1.51 (1.07 – 2.15)

SNP = single nucleotide polymorphism, MAF = minor allele frequency, OR = odds ratio, CI = confidence interval, *RARB* = retinoic acid receptor beta, *ROBO2* = roundabout axon guidance receptor homolog 2 (Drosophila), *EYA1* = eyes absent homolog 1 (Drosophila), *GFRA1* = GDNF family receptor alpha 1, *GREM1* = gremlin 1 cysteine knot superfamily homolog (Xenopus laevis), *UPK3A* = uroplakin 3A.

# top 14 p-value in the combined association results of all cases and the two endo-phenotype groups (clear cut primary VUR and duplex collecting system+VUR); SNP was analysed in stage 2 because of this result.

$/underlined five SNPs in the joint analysis showing a trend towards association (a 95% CI for the OR that was not equal to one and a p-value smaller than 0.01); warrant replication.

* CHI2 test.

** Cochran-Mantel-Haenszel test.

*** rs1403848 is in strong linkage disequilibrium (D' = 1 and r^2^> = 0.95) with rs1666130. Since rs1666130 is a perfect proxy for rs1403848, rs1403848 was not genotyped in stage two.

**** rs10103397 and rs9298164 are in strong linkage disequilibrium (D' = 1 and r^2^> = 0.95) with rs3735935. Since rs3735935 is a perfect proxy for both rs10103397 and rs9298164, they were not genotyped in stage two.

The joint results for the top 14 SNPs of the stage one and the stage two cohorts, including the analyses in the two phenotype subsets, are shown in Tabel 1. They did not reach significant p-values when corrected for multiple testing (p<8.6*10–5). Analyses of the permuted datasets did not yield significant p-values either (data not shown). The results of the stage two cohort in themselves do not replicate the stage one p-values.

Scrutinizing the joint results in the overall cohort and the two phenotype subgroups for interesting trends, revealed five SNPs (rs4476545, rs1666130, rs3735935, rs7497354 and rs1057353) and three perfect proxies (rs1403848, rs10103397, rs9298164) in four genes (*ROBO2, EYA1, GREM1, UPK3A*), that had (1) a 95% confidence interval (95% CI) for the odds ratio (OR) that was not equal to one and (2) a p-value smaller than 0.01 (Table 1).

For two genes, *GREM1* (ENSG00000166923, OR 0.77 (95% CI 0.65 – 0.91) and *ROBO2* (ENSG00000185008, OR 0.80 (95% CI 0.69 – 0.94)), it was mainly the primary VUR cases that contributed to the overall trend. For *EYA1*, (ENSG00000104313, OR 1.27 (95% CI 1.07 – 1.51)) the trend that would support our hypothesis (arbitrarily set at p<0.05), was visible in the joint results from both phenotype subgroups. The trend in the *UPK3A* gene (ENSG00000100373, OR 1.52 (95% CI 1.11 – 2.06) only showed in the subgroup with duplex collecting systems and VUR. In *ROBO2*, *EYA1* and *UPK3A,* more than one SNP showed in the best results list and linkage disequilibrium plots showing the allelic association between the SNPs are shown in Figure S1.

Although the result in *UPK3A* was not significant, it was intriguing given the limited sample size of the subgroup of duplex collecting system patients. This gene was subsequently sequenced in all duplex collecting system patients and we identified three inherited missense mutations that were not present in 96 control chromosomes. In silico analysis suggested that these amino acid substitutions have, at most, a mild effect on the protein (Table S3). In one of the parents with the mutation there is an indication of the presence of a duplex collecting system on renal ultrasound (Table S3). This family will be followed up in a separate study.

Five SNPs in three genes (*TGFB1* (ENSG00000105329), *GNB3* (ENSG00000111664) and *VEGFA* (ENSG00000112715)) were included in the stage one study because they were associated with VUR in other populations. [31]–[36] The SNP in *VEGFA* did not pass quality control criteria. Only rs1800469 in *TGFB1* showed a marginal effect in stage one (OR 1.32; 95% CI 1.03–1.70; p = 0.028) but it did not reach the threshold for inclusion in stage two.

## Discussion

A cohort of VUR patients was screened for association with tag SNPs covering 44 candidate genes (Table S1) that are related to ureteric budding function (Schedl [60]: figure 3: http://www.nature.com/nrg/journal/v8/n10/fig_tab/nrg2205_F3.html#figure-title). No significant associations were detected in this exploratory, candidate pathway association study in the Dutch population. The best results of the study show common genetic variants in *GREM1, EYA1* and *ROBO2* in the subgroup with isolated primary VUR and of genetic variants in *EYA1* and *UPK3A* in the subgroup with duplex collecting systems.

Our study had several limitations. There was 80% power to detect an effect size of >1.57 (or a protective effect of <0.64). Either we did not detect a larger (>1.57) effect (20% chance), or effects of genetic variants in ureteric budding genes are more moderate (<1.57) and therefore not significantly detected by our study, or the selected SNPs are not associated with VUR in our cohort. The study was designed before it was fully known that the effect sizes of genetic variants in complex diseases are usually lower than 1.6. But even today this would be a valid study to perform since VUR inheritance patterns most likely range from Mendelian to truely multifactorial, and variants with higher effect sizes are sometimes detected in association studies for complex diseases. [37]

It was impossible to obtain a control cohort with phenotyped controls; not only would it have been infeasible to perform a renal ultrasound in well over 1000 adults, it would also be pointless, since most VUR patients (i.e. children) grow out of VUR once they become adults. We did incorporate the 1% phenocopy rate in our power calculation. Furthermore, as was discussed by McCarthy et al, [38] in complex diseases with a prevalence of 5% or less, the increase in power gained by increasing the sample size of a population based control cohort is often larger than the increase in power gained by thoroughly phenotyping a smaller set of controls.

As always, the moment of study design signifies a snapshot of current knowledge, that is swiftly outdated. This means that some genes that are currently interesting CAKUT genes, like *FGFR2, FRS2,* [39], [40] [39], [40] ETV4 and ETV5 [41] [41] were not considered for inclusion.

The null hypothesis (no association of common genetic variants in the genes in the ureteric budding pathway) cannot be discarded based on our results. Hence, our reported findings should be interpreted cautiously and warrant replication in other, preferably larger, cohorts.

Association studies such as this, in common complex diseases, are suited to detecting common genetic variants with modest individual effect sizes. [21], [22] Earlier studies have shown that rare pathogenic mutations in three of these genes cause human urinary tract malformations or syndromes. Mutations and microdeletions of *EYA1* cause Branchiootorenal Syndrome (BOR, MIM 113650) [42] and branchiootorenal spectrum disorders. [43] Among other congenital anomalies, BOR is characterized by renal anomalies in 38.2% of mutation carriers. [44] These anomalies typically include renal agenesis, hypoplasia or dysplasia, but VUR is also part of the phenotypic spectrum. [43] *ROBO2* was shown to be mutated in a small number of (familial) VUR/CAKUT patients. [23], [29], [45] Mutations in *UPK3A* are a cause for renal adysplasia, a phenotype within the CAKUT spectrum. [46], [47] Mouse models for all four genes show phenotypes reminiscent of VUR/CAKUT. [23], [48]–[50] It is known from other diseases that different risk variants with diverse effects in the same gene can contribute to both Mendelian (syndromal) and multifactorial phenotypes. [51]

Since VUR, both with and without a duplex collecting system, can occur within the same family, the phenotypes may partly be caused by the same underlying genetic factors, as previously discussed by Kelly et al. [18] For this reason we also included cases with a duplex collecting system in our study. Nevertheless, for the analyses, we also analyzed the two extreme phenotype subgroups (i.e. clear-cut primary VUR cases and cases with a duplex collecting system and VUR) separately. In one of the four genes (*EYA1*), the joint ORs in both groups showed a trend supporting our hypothesis of contribution of common genetic variants to the genetic susceptibility for VUR.

The subgroup association analysis identified *UPK3A* as a plausible risk factor for the duplex collecting system phenotype alone. On sequencing the complete coding region of *UPK3A* in this subgroup, we identified three inherited amino acid substitutions, which may represent susceptibility alleles. Mutations in *UPK3A* [46], [47] were not detected in VUR patients so far. [52]–[54] One, albeit weak, association between VUR and a missense polymorphism in *UPK3A* has been published. [53] Future studies will reveal whether mildly pathogenic mutations and/or common genetic variants in *UPK3A* contribute to the duplex collecting system subphenotype, or also to VUR, in general.

The trends in *GREM1* and *ROBO2* in this study are mainly derived from the clear-cut primary VUR cases. Interestingly, in one of the families in which a *ROBO2* mutation was previously identified as cause of the phenotype, [23] duplex collecting systems were also part of the phenotype. Our study only had power to detect association in the duplex collecting system subgroup with common variants with a relatively large effect size. It is therefore possible that a milder effect in this subgroup from variants in *ROBO2* remained undetected.

It appears from the linkage disequilibrium (LD) plots in Figure S1a that the three SNPs in *ROBO2* that reached the cut-off for stage two of our study, might represent two independent effects. Two SNPs are part of an LD block so the likely risk factor may be a variant anywhere in that block. In *EYA1* (Figure S1b), all four SNPs are part of the same LD block. The SNPs in *UPK3A* are not in LD. The SNP (rs1057353) that shows a trend for association with the duplex collecting system phenotype is a non-synonymous coding SNP and part of an LD block, so again the likely causative locus may be anywhere in the block, or we may have picked up an effect of this specific SNP.

As a by-product of their linkage study Cordell et al. recently performed an association scan for six candidate genes, five of which we also studied in our two cohorts. [19] Two of these genes (*ROBO2* and *UPK3A)* were included in the top results in our Dutch cohort. None of the genes were significantly associated with VUR in Cordell et al.’s study. They also tested their genome-wide linkage SNP set (∼140,000 SNPs) for association with VUR. The SNPs with the most promising p-values were not located in genes related to the ureteric budding pathway; they were therefore not studied in our cohort. Other SNPs in genes in the ureteric budding pathway were not reported, but since coverage may not have been adequate, we cannot rule out that these genes play a role in that study. [19]

SNPs in three genes (*TGFB1, GNB3* and *VEGFA*) were previously shown to be associated with VUR in other populations and therefore included in our study. [31]–[36] Only rs1800469 in *TGFB1* showed a marginal trend towards association in our Dutch cases.

Implication of genes involved in the ureteric budding pathway in multifactorial, isolated primary VUR remains to be established. Based on the large body of evidence from human and mouse studies (see references in Introduction and for Table S1), we believe there is also a role for these genes in the pathogenesis of isolated VUR. Association studies in larger cohorts will elucidate the role of common genetic variants with small effect sizes. Furthermore, as shown for *ROBO2,* [23], [45] it may well be that rare as well as common genetic variants explain part of the heritability of VUR. Future targeted sequencing of these and newly identified genes and exome sequencing studies in well-characterized multiplex families as well as sporadic cases may shed light on this alternative hypothesis. [55], [56] It is also possible that common or rare genetic variants in as yet undiscovered genes in this or another pathway will prove to be key players in the development of VUR.

In conclusion, this was the first extensive association study of the ureteric budding pathway in VUR patients and controls and provides no conclusive evidence for association of common variants in genes in the ureteric budding pathway with VUR.

## Methods

### Study Design

We used a two-stage approach in which all of the designed SNPs were genotyped in the first stage. Then a number of top SNPs were chosen to be genotyped in the second stage. The joint analysis of both stages was the end result of the study. [30] In stage one, SNPs in 44 genes were genotyped in 207 unrelated cases and 554 controls. The SNPs with the 14 lowest p-values (p value cut-off: 0.005) for association in either the whole group or a subgroup were genotyped in stage two in a second cohort of 202 cases and 892 controls. Allelic association p values were calculated per stage (chi^2^ test for independence) and combined (Cochran-Mantel-Haenszel) in PLINK. [57] The datasets were also permuted 10,000 times and analyzed in PLINK. Deviations from Hardy Weinberg equilibrium in the controls were tested with a chi2 goodness-of-fit test in PLINK (cut-off: 0.001).

### Cases and Controls

The total case population consisted of 409 VUR patients of Dutch descent (see Figure 1 for detailed information). All patients were diagnosed and treated in pediatric urology clinics of the participating Dutch university medical centers. Medical records were reviewed in order to ensure the correct diagnosis of VUR.

We performed both overall and endo-phenotype analyses (Figure 1). The first endo-phenotype group consisted of clear-cut primary VUR patients, i.e. with no other mild urological findings, like mild dysfunctional voiding, a relative meatal stenosis, or insignificant urethral valves (n = 111/207 and 87/202). The second endo-phenotype group consisted of VUR patients with only complete or incomplete duplex collecting systems (n = 47/207 and 58/202).

The control group comprised two independent cohorts (Figure 1) in order to obtain a larger sample size and more power. The first were 554 healthy Dutch donors from the blood banks in Amsterdam and Utrecht. [58] The second group were 338 healthy Dutch volunteers recruited for an unrelated study on amyotrophic lateral sclerosis. [59] Controls in stage one were entirely from the blood donor group, while controls in stage two were from both groups.

All patients and controls gave their informed consent and the study was approved by the ethics review committees of each of the participating hospitals (UMC Utrecht Institutional Review Board protocol 00–103/K).

### Gene Selection

For stage one, initially 52 candidate genes were selected based on at least one of the following criteria (Table S1):

(a) direct involvement in the ureteric budding pathway as reviewed by Schedl [60]: figure 3: http://www.nature.com/nrg/journal/v8/n10/fig_tab/nrg2205_F3.html#figure-title; (b) evidence from the literature for implication in the ureteric budding pathway; (c) involvement in human syndromes associated with VUR or VUR-related phenotypes; (d) five SNPs in three genes (TGFB1, GNB3 and VEGFA) were included because they showed association with VUR or VUR-related phenotypes in other studies (the genes were not tagged, only the specific genetic variations were included for replication), (e) 8 “wildcard genes” were included that showed co-expression with the core group of candidate genes as reviewed by Schedl [60]: figure 3: http://www.nature.com/nrg/journal/v8/n10/fig_tab/nrg2205_F3.html#figure-title, in an online database of co-expression (‘Gemma’, http://www.chibi.ubc.ca/Gemma/). SNPs in 44 genes passed our quality control criteria (see ‘Quality Control’).

### SNP Selection

For stage one, 634 tag SNPs were selected with the Tagger program for the following parameters: r^2^>0.8, minor allele frequency (MAF) >0.1, pairwise or aggressive tagging. Each tagged locus included the coding part of the gene and at least 3 kb of the promoter region and 2 kb of the 3' end. If only a few tag SNPs were available at suboptimal parameters, all the known SNPs were included. Furthermore, by using FastSNP, [61] where possible we added SNPs with a predicted functional effect in the chosen genes (n = 124). This second SNP category was allowed to have a MAF <0.1. For 7 of 52 genes, there were no tagging SNPs available, so only functional SNPs were included for these (see Table S4).

### Genotyping

DNA samples for stage one were derived from whole blood. In stage two, DNA samples from cases were either derived from whole blood or Oragene saliva kits (DNA Genotek, Ottawa, Canada), but in controls they originated from whole blood. DNA of part of the stage two control samples had been previously amplified (REPLI-G, Qiagen, Valencia, CA, USA).

SNP genotyping for the discovery cohort was performed with a GoldenGate assay on an Illumina BeadStation 500GX per the manufacturer’s protocol (Illumina, San Diego, USA). Raw data were analyzed with Bead Studio software (Applied Biosystems, Nieuwerkerk a/d IJssel, the Netherlands). Clustering for all SNPs was checked manually and any dubiously clustered SNPs were removed.

Genotyping of the 14 SNPs in the replication cohort was performed with TaqMan probes and primers and an ABI 7900HT system (Applied Biosystems). Assay IDs are provided in Table S5. Clustering for all SNPs was checked manually. As it proved difficult to genotype rs1057353 satisfactorily with a TaqMan assay, it was partly genotyped via Sanger sequencing. See Table S6 for primer details. Because of linkage disequilibrium (D' = 1 and r^2^ ≥ 0.95) between rs1666130 and rs1403848 in *ROBO2* and rs3735935, rs9298164 and rs10103397 in *EYA1*, we included one SNP from each set (rs1666130 and rs3735935) for genotyping in stage two. These two SNPs were perfect proxies for the three that were not actually genotyped. We consequently genotyped 11 SNPs. LD plots for the genes that showed the best results were created with Haploview version 4.2 and based on HAPMAP CEU data.

*UPK3A* was sequenced using Sanger sequencing in the endo-phenotype subgroup of VUR patients with complete or incomplete duplex collecting systems, and 96 control chromosomes. A margin of at least 143 basepairs was observed surrounding the coding regions. See Table S6 for primer details. In silico analysis of mutations was performed with Alamut version 1.4 from Interactive Biosoftware (Rouen, France).

### Quality Control

One sample was added to each of the ten 96 well plates in stage one to check for concordance. The concordance rate over 758 SNPs was 99.9%. As stage one quality-control measures, duplicate samples were removed, sample call rate, genotype call rate and Hardy-Weinberg equilibrium (HWE) within controls were determined. Initially, 758 SNPs were included in this study. Only samples with a call rate above 90% were included in further analyses (Figure 1). SNPs with a minor allele frequency (MAF) of <0.1 (188 SNPs) or a genotyping call rate of less than 90% (64 SNPs) were excluded. Four SNPs showed strong deviation from Hardy- Weinberg equilibrium in the controls (p_HWE_ <0.001) and were discarded from further analysis. After quality control, 567 successfully genotyped SNPs were used for further analysis. For the 42 genes for which tagging SNPs were included, the median percentage of tagging SNPs passing our quality criteria was 90%. For 8 of 10 genes that had only some or all functional SNPs included, these SNPs did not pass the quality control. So effectively, SNPs in 44 genes were tested for association with VUR (see Tables S1 and S4).

For the stage two cohort, we determined sample call rate, genotype call rate, MAF and HWE. Only samples with a call rate >90% were included in further analyses (Figure 1). In stage two, all SNPs satisfied the quality control criteria (genotyping rate >90%, MAF>0.1, p_HWE_ >0.001 in controls). Four 384 well plates were used for stage two. Two of these plates contained duplicate control samples, the concordance rate for these samples was 100%. The stage two ALS control plates did not contain duplicate control samples as they were not created in our own lab.

### Power Estimation

The power to detect an effect in the joint cohorts under the assumption of an additive model was estimated using the Genetic Power Calculator [62] (Figure S2). We assumed a prevalence of 0.01, a high risk allele frequency (A) of 0.25, a disease allele frequency of 0.25 a D’ of 1, and the use of unselected controls. This study had 80% power to detect a heterozygote relative risk of 1.57 (or a protective effect of 1/1.57 = 0.64) at a significance level of 8.6*10^−5^ ( = 0.05/(567+14) tests). As can be appreciated from Figure S2, the study had approximately 4% power to detect each variant that has a heterozygote relative risk of 1.2. It would have taken a five-fold number of cases and controls to obtain 80% power to detect a 1.2 heterozygote relative risk (not shown in Figure S2).

## Supporting Information

Figure S1 **LD plots for ROBO2, EYA1 and UPK3A.** **(a)** LD plot (based on Hapmap r^2^ data) for *ROBO2* (3 kb upstream and 2 kb downstream). SNPs that reached the cut-off for stage two of our study are highlighted (from left to right: rs4476545, rs1666130 and rs1403848; also see Table 1). **(b)** LD plot (based on Hapmap r^2^ data) for *EYA1* (3 kb upstream and 2 kb downstream). SNPs that reached the cut-off for stage two of our study are highlighted (from left to right: rs10103397, rs9298164, rs3735935, rs1481800, also see Table 1). **(c)** LD plot (based on Hapmap r^2^ data) for *UPK3A* (3 kb upstream and 2 kb downstream). SNPs genotyped in stage two of our study are highlighted (also see Table 1).
(EPS)

Figure S2 **Power estimation.** This study had 80% power to detect an association with a heterozygote effect size of 1.57.
(EPS)

Table S1 **Genes selected for vesico-ureteral reflux association study.**
(DOCX)

Table S2 **567 SNPs in the VUR association study that passed our quality control criteria.**
(DOCX)

Table S3 **Three inherited** ***UPK3A*** **mutations identified in the duplex collecting system subgroup. Results of in silico analysis, online database queries, and renal ultrasound in parents.**
(DOCX)

Table S4 **Tagging and functional SNPs in this association study that passed our quality control criteria.**
(DOCX)

Table S5 **TaqMan assay IDs for SNPs genotyped in stage two (Applied Biosystems).**
(DOCX)

Table S6 **Primer sequences used for** ***UPK3A*** sequencing and sequencing of rs1057353 (indicated with *).
(DOCX)
