# Supplementary material for: Genes in the Ureteric Budding Pathway: Association Study on Vesico-Ureteral Reflux Patients
Source: PLoS One. 2012 Apr 27;7(4):e31327. doi: 10.1371/journal.pone.0031327 (PMC3338743; doi:10.1371/journal.pone.0031327)
Supplement: Table S1 — Genes selected for vesico-ureteral reflux association study. (DOCX) [file pone.0031327.s003.docx]

**Table S1.** Genes selected for vesico-ureteral reflux association study.

| HGNC symbol | MIM gene ID | chromosome, band | (a) direct involvement in the ureteric budding pathway as reviewed by Schedl ^1^ (Figure 3) | (b) evidence for implication in the ureteric budding pathway (human or mouse, genetic, functional or expression studies) | (c) implicated in human syndromes associated with VUR or other urinary tract (congenital) anomalies | (d) association of single or few genetic polymorphisms in the gene with VUR or VUR-related phenotypes (these genes were not tagged, only the specific genetic variations were included for replication) | (e) "wildcard genes" that showed co-expression with the core group of candidate genes as reviewed by Schedl ^1^ (Figure 3) according to 'Gemma' (online database of coexpression, <http://www.chibi.ubc.ca/Gemma/>) | (f) other reasons | all OMIM morbid IDs with known gene(s) that have a urinary tract congenital anomaly in "clinical synopsis" | 7 key references that review the ureteric budding / early kidney induction pathway | miscellaneous references |
| --- | --- | --- | --- | --- | --- | --- | --- | --- | --- | --- | --- |
| *AGTR2** | MIM 300034 | Xq23 |  | x |  |  |  |  |  | 4, 6 |  |
| *BMP4* | MIM 112262 | 14q22.2 | x | x |  |  |  |  | OMIM 607932;MICROPHTHALMIA, SYNDROMIC 6 | 1, 4-6 |  |
| *CTNNB1* | MIM 116806 | 3p22.1 |  |  |  |  |  | in early embryonic kidney development |  | 1 |  |
| *E2F4** | MIM 600659 | 16q22.1 |  |  |  |  | x |  |  |  |  |
| *EMX2* | MIM 600035 | 10q26.11 |  | x |  |  |  |  |  | 4-6 |  |
| *EYA1* | MIM 601653 | 8q13.3 | x | x | x |  |  |  | OMIM 113650; BRANCHIOOTORENAL SYNDROME 1 / OMIM 113620; BRANCHIOOCULOFACIAL SYNDROME; BOFS / OMIM 610896; BRANCHIOOTORENAL SYNDROME 2; BOR2 | 1-6 | 15 |
| *FGF10* | MIM 602115 | 5p12 |  |  |  |  |  | in early embryonic kidney development | OMIM 149730; LACRIMOAURICULODENTODIGITAL SYNDROME |  | 13 |
| *FGF7* | MIM 148180 | 15q21.1 |  |  |  |  |  | in early embryonic kidney development |  | 4 | 20 |
| *FOXC1* | MIM 601090 | 6p25.3 | x | x |  |  |  |  |  | 1, 3-6 |  |
| *FOXC2** | MIM 602402 | 16q24.1 |  | x | x |  |  |  |  | 4-6 | 22 |
| *FSTL1* | MIM 605547 | 3q13.33 |  |  |  |  | x |  |  |  |  |
| *GATA3* | MIM 131320 | 10p14 |  |  | x |  |  |  | OMIM 146255; HYPOPARATHYROIDISM, SENSORINEURAL DEAFNESS, AND RENAL DISEASE | 5, 6 | 16 |
| *GDF11** | MIM 603936 | 12q13.2 | x | x |  |  |  |  |  | 1, 3, 6 |  |
| *GDNF* | MIM 600837 | 5p13.2 | x | x |  |  |  |  |  | 1, 3-6, 8 |  |
| *GFRA1* | MIM 601496 | 10q25.3 | x | x |  |  |  |  |  | 1, 5, 6, 8 |  |
| *GNB3* | MIM 139130 | 12p13.31 |  |  |  | x |  |  |  | 6 | 19 |
| *GREM1* | MIM 603054 | 15q13.3 | x | x |  |  |  |  |  | 1 |  |
| *HOXA11* | MIM 142958 | 7p15.2 | x | x |  |  |  |  |  | 1-6 |  |
| *HOXC11* | MIM 605559 | 12q13.13 | x | x |  |  |  |  |  | 1-3, 5, 6 |  |
| *HOXD11* | MIM 142986 | 2q31.1 | x | x |  |  |  |  |  | 1-6 |  |
| *IGHMBP2* | MIM 600502 | 11q13.2 |  |  |  |  | x |  |  |  |  |
| *ITGA8* | MIM 604063 | 10p13 | x | x |  |  |  |  |  | 1, 4, 5 |  |
| *KIAA0241* |  | 7p14.3 |  |  |  |  | x |  |  |  |  |
| *LHX1** | MIM 601999 | 17q12 |  |  |  |  |  | in early embryonic kidney development |  | 1, 4, 5 |  |
| *MXRA8** |  | 1p36.33 |  |  |  |  | x |  |  |  |  |
| *NPNT* | MIM 610306 | 4q24 | x | x |  |  |  |  |  | 1 |  |
| *OSR1* | MIM 608891 | 2p24.1 |  | x |  |  |  |  |  | 1, 2 |  |
| *PAX2* | MIM 167409 | 10q24.31 | x | x | x |  |  |  | OMIM 120330; PAPILLORENAL SYNDROME | 1-6, 8 | 17 |
| *RARA* | MIM 180240 | 17q21.2 |  | x |  |  |  |  | OMIM 182290; SMITH-MAGENIS SYNDROME | 4-6 | 7 |
| *RARB* | MIM 180220 | 3p24.2 |  | x |  |  |  |  |  | 4-6 | 7 |
| *RARG* | MIM 180190 | 12q13.13 |  | x |  |  |  |  |  | 4 | 7 |
| *RET* | MIM 164761 | 10q11.21 | x | x |  |  |  |  |  | 1-6, 8 |  |
| *ROBO2* | MIM 602431 | 3p12.3 | x | x |  |  |  |  | OMIM 610878; VESICOURETERAL REFLUX 2 | 1, 5, 6, 8 | 9, 10 |
| *SALL1* | MIM 602218 | 16q12.1 | x | x | x |  |  |  | OMIM 107480; TOWNES-BROCKS SYNDROME | 1, 3-6 | 21 |
| *SIX1** | MIM 601205 | 14q23.1 |  | x | x |  |  |  | OMIM 608389; BRANCHIOOTIC SYNDROME 3 | 1-3, 5, 6 | 15 |
| *SIX2* | MIM 604994 | 2p21 |  | x |  |  |  |  |  | 1-3 |  |
| *SLIT2* | MIM 603746 | 4p15.31 | x | x |  |  |  |  |  | 1, 5, 6, 8 |  |
| *SPRY1* | MIM 602465 | 4q28.1 | x | x |  |  |  |  |  | 1, 6, 8 |  |
| *SPRY2* | MIM 602466 | 13q31.1 |  | x |  |  |  |  |  | 8 |  |
| *TGFB1* | MIM 190180 | 19q13.2 |  |  |  | x |  |  |  | 4, 6 | 14, 23, 24 |
| *THRA* | MIM 190120 | 17q21.1 |  |  |  |  | x |  |  |  |  |
| *TNRC6B* | MIM 610740 | 22q13.1 |  |  |  |  | x |  |  |  |  |
| *UPK1A* | MIM 611557 | 19q13.12 |  |  |  |  |  | in urothelial plaque with UPK2 and 3A |  |  | 11, 12 |
| *UPK1B* | MIM 602380 | 3q13.32 |  |  |  |  |  | in urothelial plaque with UPK2 and 3A |  |  | 11, 12 |
| *UPK2* | MIM 611558 | 11q23.3 |  | x |  |  |  |  |  | 6 | 11, 12 |
| *UPK3A* | MIM 611559 | 22q13.31 |  | x |  |  |  |  | OMIM 191830; RENAL ADYSPLASIA | 6 | 11, 12 |
| *UPK3B* | MIM 611887 | 7q11.23 |  |  |  |  |  | in urothelial plaque with UPK2 and 3A |  |  | 11, 12 |
| *VEGFA** | MIM 192240 | 6p21.1 |  |  |  | x |  |  |  | 4 | 14 |
| *WNT11* | MIM 603699 | 11q13.5 |  |  |  |  |  | in early embryonic kidney development |  | 1, 5 |  |
| *WNT9B* | MIM 602864 | 17q21.32 |  | x |  |  |  | in early embryonic kidney development |  | 1 |  |
| *WT1* | MIM 607102 | 11p13 | x | x | x |  |  |  | OMIM 194080; DENYS-DRASH SYNDROME / OMIM 136680; FRASIER SYNDROME / OMIM 137357; GENITOURINARY DYSPLASIA COMPONENT OF WAGR SYNDROME / OMIM 256370; NEPHROTIC SYNDROME, EARLY-ONSET, WITH DIFFUSE MESANGIAL SCLEROSIS / OMIM 194070; WILMS TUMOR 1 / OMIM 194072; WAGR SYNDROME / OMIM 256370; NEPHROTIC SYNDROME, EARLY-ONSET, WITH DIFFUSE MESANGIAL SCLEROSIS | 1-6 | 18 |
| *ZIC1* | MIM 600470 | 3q24 |  |  |  |  | x |  |  |  |  |

* SNPs in these genes (functional SNPs, since no tagging SNPs were available) did not pass the quality control criteria. These genes were therefore not included in the analysis, but they were part of our initial selection of candidate genes.

(a) to (f) denote selection criteria.

**References for Supporting Table 1**

1. Schedl A: Renal abnormalities and their developmental origin. *Nature Reviews Genetics* 8: 791-802, 2007

2. Gong KQ, Yallowitz AR, Sun H, Dressler GR, Wellik DM: A hox-eya-pax complex regulates early kidney developmental gene expression. *Mol Cell Biol* 27: 7661-7668, 2007

3. Brodbeck S, & Englert C: Genetic determination of nephrogenesis: The Pax/Eya/Six gene network. *Pediatr Nephrol* 19: 249-55, 2004

4. Woolf AS, & Winyard PJ: Molecular mechanisms of human embryogenesis: Developmental pathogenesis of renal tract malformations. *Pediatr Dev Pathol* 5: 108-29, 2002

5. Bouchard M: Transcriptional control of kidney development. *Differentiation* 72: 295-306, 2004

6. Murawski IJ, & Gupta IR: Vesicoureteric reflux and renal malformations: A developmental problem. *Clin Genet* 69: 105-17, 2006

7. Mendelsohn C, Lohnes D, Decimo D, Lufkin T, LeMeur M, Chambon P, Mark M: Function of the retinoic acid receptors (RARs) during development (II). multiple abnormalities at various stages of organogenesis in RAR double mutants. *Development* 120: 2749-2771, 1994

8. Basson MA, Akbulut S, Watson-Johnson J, Simon R, Carroll TJ, Shakya R, Gross I, Martin GR, Lufkin T, McMahon AP, Wilson PD, Costantini FD, Mason IJ, Licht JD: Sprouty1 is a critical regulator of GDNF/RET-mediated kidney induction. *Dev Cell* 8: 229-39, 2005

9. Lu W, van Eerde AM, Fan X, Quintero-Rivera F, Kulkarni S, Ferguson H, Kim HG, Fan Y, Xi Q, Li QG, Sanlaville D, Andrews W, Sundaresan V, Bi W, Yan J, Giltay JC, Wijmenga C, de Jong TP, Feather SA, Woolf AS, Rao Y, Lupski JR, Eccles MR, Quade BJ, Gusella JF, Morton CC, Maas RL: Disruption of ROBO2 is associated with urinary tract anomalies and confers risk of vesicoureteral reflux. *Am J Hum Genet* 80: 616-32, 2007

10. Conte ML, Bertoli-Avella AM, de Graaf BM, Punzo F, Lama G, La Manna A, Grassia C, Rambaldi PF, Oostra BA, Perrotta S: A genome search for primary vesicoureteral reflux shows further evidence for genetic heterogeneity. *Pediatric Nephrology* 23: 587-595, 2008

11. Deng FM, Liang FX, Tu L, Resing KA, Hu P, Supino M, Hu CC, Zhou G, Ding M, Kreibich G, Sun TT: Uroplakin IIIb, a urothelial differentiation marker, dimerizes with uroplakin ib as an early step of urothelial plaque assembly. *J Cell Biol* 159: 685-94, 2002

12. Hu P, Deng FM, Liang FX, Hu CM, Auerbach AB, Shapiro E, Wu XR, Kachar B, Sun TT: Ablation of uroplakin III gene results in small urothelial plaques, urothelial leakage, and vesicoureteral reflux. *J Cell Biol* 151: 961-72, 2000

13. Ohuchi H, Hori Y, Yamasaki M, Harada H, Sekine K, Kato S, Itoh N: FGF10 acts as a major ligand for FGF receptor 2 IIIb in mouse multi-organ development. *Biochem Biophys Res Commun* 277: 643-649, 2000

14. Yim HE, Bae IS, Yoo KH, Hong YS, Lee JW: Genetic control of VEGF and TGF-β1 gene polymorphisms in childhood urinary tract infection and vesicoureteral reflux. *Pediatric Research* 62: 183-187, 2007

15. Smith RJH: Branchiootorenal Spectrum Disorders - GeneReviews. in: GeneReviews at GeneTests: Medical genetics information resource (database online). Last Updated 8/27/2009

16. Van Esch H, Groenen P, Nesbit MA, Schuffenhauer S, Lichtner P, Vanderlinden G, Harding B, Beetz R, Bilous RW, Holdaway I, Shaw NJ, Fryns JP, Van de Ven W, Thakker RV, Devriendt K: GATA3 haplo-insufficiency causes human HDR syndrome. *Nature* 406: 419-422, 2000

17. Schimmenti LA, & Eccles MR: Renal coloboma syndrome. in: GeneReviews at GeneTests: Medical genetics information resource (database online).

18. Dome JS, & Huff V: Wilms tumor overview. in: GeneReviews at GeneTests: Medical genetics information resource (database online).

19. Zagradisnik B, Bracic K, Varda NM, Vokac NK, Gregoric A: G-protein β3 subunit gene C825T polymorphism in patients with vesico-ureteric reflux. *Annales De Genetique* 47: 209-216, 2004

20. Qiao J, Uzzo R, Obara-Ishihara T, Degenstein L, Fuchs E, Herzlinger D: FGF-7 modulates ureteric bud growth and nephron number in the developing kidney. *Development* 126: 547-554, 1999

21. Kohlhase J: Townes-brocks syndrome. in: GeneReviews at GeneTests: Medical genetics information resource (database online).

22. Mansour S, Brice GW, Jeffery S, Mortimer P: Lymphedema-distichiasis syndrome. in: GeneReviews at GeneTests: Medical genetics information resource (database online).

23. Kuroda S, Solari V, Puri P: Association of transforming growth factor-β1 gene polymorphism with familial vesicoureteral reflux.*Journal of Urology* 178: 1650-1653, 2007

24. Solari V, Owen D, Puri P, Thomas D: Association of transforming growth factor-β1 gene polymorphism with reflux nephropathy. *Journal of Urology* 174: 1609-1611, 2005
