# Supplementary material for: Genes in the Ureteric Budding Pathway: Association Study on Vesico-Ureteral Reflux Patients
Source: PLoS One. 2012 Apr 27;7(4):e31327. doi: 10.1371/journal.pone.0031327 (PMC3338743; doi:10.1371/journal.pone.0031327)
Supplement: Table S2 — 567 SNPs in the VUR association study that passed our quality control criteria. (DOCX) [file pone.0031327.s004.docx]

**Table S2.** 567 SNPs in the VUR association study that passed our quality control criteria.

| SNP | gene | Chr | Illumina ID |
| --- | --- | --- | --- |
| rs13011502 | *OSR1* | 2 | rs13011502-127_B_R_1500219473 |
| rs5013667 | *OSR1* | 2 | rs5013667-127_T_F_1503075764 |
| rs1900000 | *OSR1* | 2 | rs1900000-127_B_F_1526341739 |
| rs2290030 | *SIX2* | 2 | rs2290030-127_T_R_1526341992 |
| rs921117 | *SIX2* | 2 | rs921117-127_B_R_1502489506 |
| rs847148 | *HOXD11* | 2 | rs847148-127_B_F_1503918569 |
| rs847146 | *HOXD11* | 2 | rs847146-127_B_F_1503961555 |
| rs12152294 | *RARB* | 3 | rs12152294-127_B_R_1527437869 |
| rs978142 | *RARB* | 3 | rs978142-127_B_R_1528547463 |
| rs931697 | *RARB* | 3 | rs931697-127_T_F_1528547460 |
| rs1499644 | *RARB* | 3 | rs1499644-127_B_R_1528547501 |
| rs2363594 | *RARB* | 3 | rs2363594-127_T_F_1527437876 |
| rs6765179 | *RARB* | 3 | rs6765179-127_T_F_1527437918 |
| rs6780105 | *RARB* | 3 | rs6780105-127_B_R_1528547491 |
| rs1483851 | *RARB* | 3 | rs1483851-127_B_F_1527437867 |
| rs13077354 | *RARB* | 3 | rs13077354-127_T_R_1527437894 |
| rs1872144 | *RARB* | 3 | rs1872144-127_T_R_1527437864 |
| rs322679 | *RARB* | 3 | rs322679-127_B_R_1527437901 |
| rs322677 | *RARB* | 3 | rs322677-127_B_R_1528547489 |
| rs6810082 | *RARB* | 3 | rs6810082-127_B_R_1527437911 |
| rs977224 | *RARB* | 3 | rs977224-127_T_R_1527437896 |
| rs2363596 | *RARB* | 3 | rs2363596-127_T_R_1528547494 |
| rs322668 | *RARB* | 3 | rs322668-127_B_F_1527437885 |
| rs4681045 | *RARB* | 3 | rs4681045-127_T_F_1500007022 |
| rs17016178 | *RARB* | 3 | rs17016178-127_T_R_1528547456 |
| rs1561116 | *RARB* | 3 | rs1561116-127_B_R_1527437865 |
| rs6550962 | *RARB* | 3 | rs6550962-127_T_F_1527437924 |
| rs4681047 | *RARB* | 3 | rs4681047-127_B_R_1527437889 |
| rs1483831 | *RARB* | 3 | rs1483831-127_T_R_1527437874 |
| rs6550967 | *RARB* | 3 | rs6550967-127_B_F_1527437891 |
| rs9310778 | *RARB* | 3 | rs9310778-127_B_R_1526505349 |
| rs7614710 | *RARB* | 3 | rs7614710-127_B_R_1528547465 |
| rs2195919 | *RARB* | 3 | rs2195919-127_B_R_1528547457 |
| rs7628351 | *RARB* | 3 | rs7628351-127_B_F_1528547461 |
| rs11714772 | *RARB* | 3 | rs11714772-127_B_F_1528547467 |
| rs1843028 | *RARB* | 3 | rs1843028-127_B_F_1527437861 |
| rs12330681 | *RARB* | 3 | rs12330681-127_B_F_1528547499 |
| rs2116701 | *RARB* | 3 | rs2116701-127_T_F_1527437922 |
| rs6550971 | *RARB* | 3 | rs6550971-127_T_R_1528547454 |
| rs4395373 | *RARB* | 3 | rs4395373-127_T_R_1527437920 |
| rs6783726 | *RARB* | 3 | rs6783726-127_B_R_1527437899 |
| rs4681060 | *RARB* | 3 | rs4681060-127_T_R_1527437898 |
| rs7620529 | *RARB* | 3 | rs7620529-127_B_R_1529565199 |
| rs1483827 | *RARB* | 3 | rs1483827-127_T_R_1529565198 |
| rs6805482 | *RARB* | 3 | rs6805482-127_T_F_1527345492 |
| rs6778608 | *RARB* | 3 | rs6778608-127_T_R_1527345938 |
| rs17016408 | *RARB* | 3 | rs17016408-127_B_R_1527345783 |
| rs922939 | *RARB* | 3 | rs922939-127_T_R_1527345508 |
| rs12630664 | *RARB* | 3 | rs12630664-127_T_R_1527345584 |
| rs755661 | *RARB* | 3 | rs755661-127_T_R_1526093680 |
| rs7620632 | *RARB* | 3 | rs7620632-127_B_F_1527345475 |
| rs9871002 | *RARB* | 3 | rs9871002-127_B_F_1526103479 |
| rs6550975 | *RARB* | 3 | rs6550975-127_T_R_1527345764 |
| rs6800566 | *RARB* | 3 | rs6800566-127_T_F_1527345736 |
| rs4681063 | *RARB* | 3 | rs4681063-127_T_R_1527345526 |
| rs6777544 | *RARB* | 3 | rs6777544-127_T_R_1527346184 |
| rs6550980 | *RARB* | 3 | rs6550980-127_T_F_1500538390 |
| rs6793694 | *RARB* | 3 | rs6793694-127_B_R_1526093919 |
| rs7629902 | *RARB* | 3 | rs7629902-127_B_R_1527345485 |
| rs6550981 | *RARB* | 3 | rs6550981-127_T_R_1527346364 |
| rs7616467 | *RARB* | 3 | rs7616467-127_T_R_1527345920 |
| rs2033447 | *RARB* | 3 | rs2033447-127_T_R_1527345440 |
| rs4681064 | *RARB* | 3 | rs4681064-127_B_F_1527345773 |
| rs871963 | *RARB* | 3 | rs871963-127_B_F_1503919236 |
| rs7629478 | *RARB* | 3 | rs7629478-127_T_R_1526101754 |
| rs4607073 | *RARB* | 3 | rs4607073-127_T_R_1526099952 |
| rs11715516 | *RARB* | 3 | rs11715516-127_T_F_1526090716 |
| rs6775425 | *RARB* | 3 | rs6775425-127_T_R_1526101752 |
| rs10212330 | *RARB* | 3 | rs10212330-127_B_R_1527346235 |
| rs6796669 | *RARB* | 3 | rs6796669-127_B_R_1526101739 |
| rs1286654 | *RARB* | 3 | rs1286654-127_T_R_1526091672 |
| rs1299407 | *RARB* | 3 | rs1299407-127_T_F_1527345582 |
| rs1997352 | *RARB* | 3 | rs1997352-127_B_R_1527345725 |
| rs1406575 | *RARB* | 3 | rs1406575-127_B_R_1527345643 |
| rs1153584 | *RARB* | 3 | rs1153584-127_B_F_1527345515 |
| rs1881706 | *RARB* | 3 | rs1881706-127_T_R_1527345560 |
| rs1286750 | *RARB* | 3 | rs1286750-127_B_R_1527346037 |
| rs1153589 | *RARB* | 3 | rs1153589-127_B_R_1500539600 |
| rs1153591 | *RARB* | 3 | rs1153591-127_B_R_1503288678 |
| rs1286756 | *RARB* | 3 | rs1286756-127_B_R_1503309332 |
| rs13099641 | *RARB* | 3 | rs13099641-127_T_R_1527346344 |
| rs17525900 | *RARB* | 3 | rs17525900-127_T_R_1527345944 |
| rs1153606 | *RARB* | 3 | rs1153606-127_B_R_1527345617 |
| rs1286665 | *RARB* | 3 | rs1286665-127_T_R_1527345552 |
| rs1881703 | *RARB* | 3 | rs1881703-127_T_F_1503260937 |
| rs7616062 | *RARB* | 3 | rs7616062-127_T_R_1527346290 |
| rs1286772 | *RARB* | 3 | rs1286772-127_T_R_1500536398 |
| rs1286769 | *RARB* | 3 | rs1286769-127_T_R_1500537809 |
| rs17016718 | *RARB* | 3 | rs17016718-127_T_R_1527346228 |
| rs1286761 | *RARB* | 3 | rs1286761-127_B_R_1503289171 |
| rs10510568 | *RARB* | 3 | rs10510568-127_T_F_1527345524 |
| rs17016773 | *RARB* | 3 | rs17016773-127_T_R_1527346008 |
| rs17016778 | *RARB* | 3 | rs17016778-127_T_F_1527345532 |
| rs17016781 | *RARB* | 3 | rs17016781-127_T_F_1527345622 |
| rs1286729 | *RARB* | 3 | rs1286729-127_B_F_1526088869 |
| rs1286738 | *RARB* | 3 | rs1286738-127_B_F_1527345519 |
| rs1656463 | *RARB* | 3 | rs1656463-127_T_F_1503032738 |
| rs1730221 | *RARB* | 3 | rs1730221-127_B_F_1527346165 |
| rs9809535 | *RARB* | 3 | rs9809535-127_B_R_1527345555 |
| rs4681028 | *RARB* | 3 | rs4681028-127_T_R_1527346272 |
| rs7620852 | *RARB* | 3 | rs7620852-127_T_R_1527346124 |
| rs7621140 | *RARB* | 3 | rs7621140-127_B_F_1527346031 |
| rs2164360 | *RARB* | 3 | rs2164360-127_B_F_1503361240 |
| rs1798802 | *CTNNB1* | 3 | rs1798802-127_T_F_1526233190 |
| rs4135385 | *CTNNB1* | 3 | rs4135385-127_B_R_1503910162 |
| rs3923745 | *ROBO2* | 3 | rs3923745-127_B_R_1528580403 |
| rs6784307 | *ROBO2* | 3 | rs6784307-127_B_R_1503326009 |
| rs3934881 | *ROBO2* | 3 | rs3934881-127_T_R_1528583630 |
| rs11712939 | *ROBO2* | 3 | rs11712939-127_B_R_1529565165 |
| rs6779154 | *ROBO2* | 3 | rs6779154-127_T_R_1500382203 |
| rs4476545 | *ROBO2* | 3 | rs4476545-127_B_F_1529565179 |
| rs7640155 | *ROBO2* | 3 | rs7640155-127_T_R_1500225375 |
| rs4459934 | *ROBO2* | 3 | rs4459934-127_B_R_1529565273 |
| rs7628843 | *ROBO2* | 3 | rs7628843-127_T_F_1500225374 |
| rs4624600 | *ROBO2* | 3 | rs4624600-127_T_F_1500035705 |
| rs9859970 | *ROBO2* | 3 | rs9859970-127_B_F_1500225605 |
| rs13322517 | *ROBO2* | 3 | rs13322517-127_B_R_1528580543 |
| rs4645165 | *ROBO2* | 3 | rs4645165-127_B_F_1500029520 |
| rs4475074 | *ROBO2* | 3 | rs4475074-127_B_R_1529565243 |
| rs12496474 | *ROBO2* | 3 | rs12496474-127_T_F_1529565264 |
| rs7618319 | *ROBO2* | 3 | rs7618319-127_B_F_1529565171 |
| rs4077419 | *ROBO2* | 3 | rs4077419-127_T_F_1500029459 |
| rs7432300 | *ROBO2* | 3 | rs7432300-127_B_F_1500225684 |
| rs13097139 | *ROBO2* | 3 | rs13097139-127_B_R_1529565257 |
| rs9852548 | *ROBO2* | 3 | rs9852548-127_B_R_1500034878 |
| rs11709672 | *ROBO2* | 3 | rs11709672-127_B_F_1500027429 |
| rs4684028 | *ROBO2* | 3 | rs4684028-127_T_F_1500224927 |
| rs7432274 | *ROBO2* | 3 | rs7432274-127_B_R_1500225318 |
| rs12487684 | *ROBO2* | 3 | rs12487684-127_T_F_1500020771 |
| rs17769969 | *ROBO2* | 3 | rs17769969-127_B_R_1528582541 |
| rs6772736 | *ROBO2* | 3 | rs6772736-127_B_R_1503961664 |
| rs12491865 | *ROBO2* | 3 | rs12491865-127_B_R_1500381684 |
| rs879728 | *ROBO2* | 3 | rs879728-127_T_R_1529565174 |
| rs4072806 | *ROBO2* | 3 | rs4072806-127_B_R_1500224845 |
| rs7430171 | *ROBO2* | 3 | rs7430171-127_T_R_1500029853 |
| rs1470203 | *ROBO2* | 3 | rs1470203-127_T_F_1528582876 |
| rs17822079 | *ROBO2* | 3 | rs17822079-127_B_F_1529565185 |
| rs6776288 | *ROBO2* | 3 | rs6776288-127_T_R_1529565254 |
| rs13087163 | *ROBO2* | 3 | rs13087163-127_B_R_1529565147 |
| rs11928406 | *ROBO2* | 3 | rs11928406-127_T_F_1529565266 |
| rs998037 | *ROBO2* | 3 | rs998037-127_B_F_1500025182 |
| rs9873219 | *ROBO2* | 3 | rs9873219-127_T_F_1529565270 |
| rs12637117 | *ROBO2* | 3 | rs12637117-127_B_F_1529565245 |
| rs9820365 | *ROBO2* | 3 | rs9820365-127_T_R_1502589545 |
| rs1978940 | *ROBO2* | 3 | rs1978940-127_B_R_1500020996 |
| rs2028512 | *ROBO2* | 3 | rs2028512-127_B_F_1500035439 |
| rs9818075 | *ROBO2* | 3 | rs9818075-127_T_F_1529565122 |
| rs10511055 | *ROBO2* | 3 | rs10511055-127_B_R_1528581879 |
| rs7642312 | *ROBO2* | 3 | rs7642312-127_T_F_1500382260 |
| rs9869100 | *ROBO2* | 3 | rs9869100-127_B_R_1500028794 |
| rs6767250 | *ROBO2* | 3 | rs6767250-127_T_R_1529565194 |
| rs4683972 | *ROBO2* | 3 | rs4683972-127_T_R_1529565190 |
| rs9872037 | *ROBO2* | 3 | rs9872037-127_T_F_1500034945 |
| rs6768736 | *ROBO2* | 3 | rs6768736-127_T_F_1500382657 |
| rs6548505 | *ROBO2* | 3 | rs6548505-127_B_R_1528583803 |
| rs9836971 | *ROBO2* | 3 | rs9836971-127_B_R_1500222332 |
| rs7639939 | *ROBO2* | 3 | rs7639939-127_B_R_1529565247 |
| rs9883718 | *ROBO2* | 3 | rs9883718-127_T_F_1529565160 |
| rs12492221 | *ROBO2* | 3 | rs12492221-127_T_F_1500020785 |
| rs12490828 | *ROBO2* | 3 | rs12490828-127_T_F_1529565250 |
| rs9830337 | *ROBO2* | 3 | rs9830337-127_T_F_1500024965 |
| rs6777631 | *ROBO2* | 3 | rs6777631-127_T_F_1500225661 |
| rs6781059 | *ROBO2* | 3 | rs6781059-127_B_R_1529565195 |
| rs7652402 | *ROBO2* | 3 | rs7652402-127_B_F_1500382268 |
| rs7651943 | *ROBO2* | 3 | rs7651943-127_T_R_1502519293 |
| rs2121817 | *ROBO2* | 3 | rs2121817-127_T_R_1529565252 |
| rs2166802 | *ROBO2* | 3 | rs2166802-127_T_R_1529565260 |
| rs7614439 | *ROBO2* | 3 | rs7614439-127_T_R_1500225368 |
| rs12487172 | *ROBO2* | 3 | rs12487172-127_B_R_1503369132 |
| rs13064369 | *ROBO2* | 3 | rs13064369-127_B_F_1500224372 |
| rs6799832 | *ROBO2* | 3 | rs6799832-127_T_F_1503107230 |
| rs935526 | *ROBO2* | 3 | rs935526-127_B_F_1500382450 |
| rs1447846 | *ROBO2* | 3 | rs1447846-127_T_F_1503022897 |
| rs1666130 | *ROBO2* | 3 | rs1666130-127_B_R_1500381425 |
| rs1403848 | *ROBO2* | 3 | rs1403848-127_B_R_1500041425 |
| rs1721175 | *ROBO2* | 3 | rs1721175-127_T_R_1500033884 |
| rs876675 | *ROBO2* | 3 | rs876675-127_B_R_1501188725 |
| rs10779982 | *ROBO2* | 3 | rs10779982-127_B_R_1500020609 |
| rs1839795 | *ROBO2* | 3 | rs1839795-127_B_F_1500381564 |
| rs1031377 | *ROBO2* | 3 | rs1031377-127_T_R_1500020587 |
| rs9855251 | *UPK1B* | 3 | rs9855251-127_T_R_1500022016 |
| rs13079334 | *UPK1B* | 3 | rs13079334-127_T_R_1526361414 |
| rs2903301 | *UPK1B* | 3 | rs2903301-127_T_R_1526361752 |
| rs9832079 | *UPK1B* | 3 | rs9832079-127_B_R_1526361431 |
| rs17281535 | *UPK1B* | 3 | rs17281535-127_B_F_1526361797 |
| rs4234654 | *UPK1B* | 3 | rs4234654-127_T_F_1500024231 |
| rs7613994 | *UPK1B* | 3 | rs7613994-127_B_R_1526361979 |
| rs9814507 | *UPK1B* | 3 | rs9814507-127_B_F_1526361765 |
| rs6797198 | *UPK1B* | 3 | rs6797198-127_T_R_1500385632 |
| rs6784773 | *UPK1B* | 3 | rs6784773-127_T_R_1526361772 |
| rs4455314 | *UPK1B* | 3 | rs4455314-127_B_F_1526361821 |
| rs10934486 | *UPK1B* | 3 | rs10934486-127_B_R_1500027353 |
| rs6772525 | *UPK1B* | 3 | rs6772525-127_T_R_1526361216 |
| rs9878996 | *UPK1B* | 3 | rs9878996-127_B_F_1526361355 |
| rs7628485 | *UPK1B* | 3 | rs7628485-127_B_R_1526361383 |
| rs13098659 | *UPK1B* | 3 | rs13098659-127_T_F_1526361226 |
| rs7622158 | *UPK1B* | 3 | rs7622158-127_T_R_1500220725 |
| rs1700 | *FSTL1* | 3 | rs1700-127_T_F_1500033881 |
| rs1259327 | *FSTL1* | 3 | rs1259327-127_T_F_1500524844 |
| rs1147695 | *FSTL1* | 3 | rs1147695-127_T_F_1529245602 |
| rs1147700 | *FSTL1* | 3 | rs1147700-127_B_R_1528587787 |
| rs4676778 | *FSTL1* | 3 | rs4676778-127_B_R_1500041841 |
| rs1147702 | *FSTL1* | 3 | rs1147702-127_B_R_1529246877 |
| rs13097755 | *FSTL1* | 3 | rs13097755-127_B_F_1500220955 |
| rs1259294 | *FSTL1* | 3 | rs1259294-127_B_F_1500020803 |
| rs1259299 | *FSTL1* | 3 | rs1259299-127_T_R_1500027545 |
| rs1402372 | *FSTL1* | 3 | rs1402372-127_T_R_1529246900 |
| rs9879383 | *FSTL1* | 3 | rs9879383-127_T_R_1529248534 |
| rs1259329 | *FSTL1* | 3 | rs1259329-127_T_R_1500384338 |
| rs13326852 | *FSTL1* | 3 | rs13326852-127_B_F_1529246895 |
| rs9833875 | *ZIC1* | 3 | rs9833875-127_T_F_1500211889 |
| rs564041 | *SLIT2* | 4 | rs564041-127_T_R_1503244372 |
| rs7655084 | *SLIT2* | 4 | rs7655084-127_T_R_1503308195 |
| rs17534458 | *SLIT2* | 4 | rs17534458-127_T_F_1529565234 |
| rs500869 | *SLIT2* | 4 | rs500869-127_T_F_1529565192 |
| rs6447952 | *SLIT2* | 4 | rs6447952-127_B_R_1503294884 |
| rs7680377 | *SLIT2* | 4 | rs7680377-127_T_R_1503107972 |
| rs1323062 | *SLIT2* | 4 | rs1323062-127_T_F_1503348958 |
| rs13435197 | *SLIT2* | 4 | rs13435197-127_T_F_1529565168 |
| rs12646142 | *SLIT2* | 4 | rs12646142-127_B_F_1500418530 |
| rs4696953 | *SLIT2* | 4 | rs4696953-127_B_R_1529565203 |
| rs10938795 | *SLIT2* | 4 | rs10938795-127_B_F_1529564983 |
| rs10938796 | *SLIT2* | 4 | rs10938796-127_B_R_1529565205 |
| rs6447955 | *SLIT2* | 4 | rs6447955-127_T_F_1503324475 |
| rs4406013 | *SLIT2* | 4 | rs4406013-127_B_F_1529565221 |
| rs4552454 | *SLIT2* | 4 | rs4552454-127_T_F_1503373648 |
| rs13142826 | *SLIT2* | 4 | rs13142826-127_B_R_1500189167 |
| rs9996157 | *SLIT2* | 4 | rs9996157-127_T_R_1500189561 |
| rs17537997 | *SLIT2* | 4 | rs17537997-127_B_R_1529565213 |
| rs16869595 | *SLIT2* | 4 | rs16869595-127_T_F_1529565182 |
| rs6849536 | *SLIT2* | 4 | rs6849536-127_T_R_1502557447 |
| rs11933090 | *SLIT2* | 4 | rs11933090-127_T_F_1529565210 |
| rs884770 | *SLIT2* | 4 | rs884770-127_T_F_1528585856 |
| rs11733205 | *SLIT2* | 4 | rs11733205-127_B_F_1500001926 |
| rs1033111 | *SLIT2* | 4 | rs1033111-127_T_R_1503348581 |
| rs491795 | *SLIT2* | 4 | rs491795-127_B_F_1500364352 |
| rs11932685 | *SLIT2* | 4 | rs11932685-127_B_R_1529565225 |
| rs17612037 | *SLIT2* | 4 | rs17612037-127_T_F_1528586114 |
| rs525684 | *SLIT2* | 4 | rs525684-127_T_F_1529565224 |
| rs12374415 | *SLIT2* | 4 | rs12374415-127_T_R_1529564982 |
| rs490478 | *SLIT2* | 4 | rs490478-127_B_R_1502496707 |
| rs556816 | *SLIT2* | 4 | rs556816-127_B_F_1502558120 |
| rs6825654 | *SLIT2* | 4 | rs6825654-127_B_R_1500190014 |
| rs519813 | *SLIT2* | 4 | rs519813-127_T_F_1503278111 |
| rs16869706 | *SLIT2* | 4 | rs16869706-127_T_F_1529564988 |
| rs7690660 | *SLIT2* | 4 | rs7690660-127_B_F_1502509622 |
| rs3775815 | *SLIT2* | 4 | rs3775815-127_B_F_1502546164 |
| rs2168801 | *SLIT2* | 4 | rs2168801-127_T_F_1529564994 |
| rs2322557 | *SLIT2* | 4 | rs2322557-127_B_R_1529565187 |
| rs573118 | *SLIT2* | 4 | rs573118-127_B_F_1503364183 |
| rs3775816 | *SLIT2* | 4 | rs3775816-127_T_R_1503293615 |
| rs3775820 | *SLIT2* | 4 | rs3775820-127_B_R_1528593929 |
| rs675465 | *SLIT2* | 4 | rs675465-127_B_R_1529565207 |
| rs7690492 | *SLIT2* | 4 | rs7690492-127_T_R_1529564976 |
| rs7669094 | *SLIT2* | 4 | rs7669094-127_T_R_1529565232 |
| rs3775824 | *SLIT2* | 4 | rs3775824-127_T_R_1529565228 |
| rs649424 | *SLIT2* | 4 | rs649424-127_T_R_1529565230 |
| rs2292439 | *SLIT2* | 4 | rs2292439-127_T_F_1503103810 |
| rs10516357 | *SLIT2* | 4 | rs10516357-127_B_R_1500189046 |
| rs506358 | *SLIT2* | 4 | rs506358-127_B_F_1502590393 |
| rs644607 | *SLIT2* | 4 | rs644607-127_T_R_1502507922 |
| rs7666974 | *SLIT2* | 4 | rs7666974-127_B_F_1503315095 |
| rs2168802 | *SLIT2* | 4 | rs2168802-127_B_F_1503370986 |
| rs17620814 | *SLIT2* | 4 | rs17620814-127_T_F_1529565218 |
| rs17621372 | *SLIT2* | 4 | rs17621372-127_B_F_1500419020 |
| rs1379659 | *SLIT2* | 4 | rs1379659-127_B_R_1502542514 |
| rs6823809 | *NPNT* | 4 | rs6823809-127_B_F_1526341403 |
| rs4600917 | *NPNT* | 4 | rs4600917-127_T_R_1503040448 |
| rs35132891 | *NPNT* | 4 | rs35132891-127_T_F_1526341374 |
| rs7677557 | *NPNT* | 4 | rs7677557-127_B_F_1526341407 |
| rs7680832 | *NPNT* | 4 | rs7680832-127_T_F_1526341338 |
| rs6817700 | *NPNT* | 4 | rs6817700-127_B_R_1503917505 |
| rs159762 | *SPRY1* | 4 | rs159762-127_B_R_1527230141 |
| rs300574 | *SPRY1* | 4 | rs300574-127_B_F_1503925150 |
| rs3749692 | *GDNF* | 5 | rs3749692-127_T_F_1529565048 |
| rs7731209 | *GDNF* | 5 | rs7731209-127_B_F_1529565001 |
| rs2973050 | *GDNF* | 5 | rs2973050-127_T_R_1528595520 |
| rs1549250 | *GDNF* | 5 | rs1549250-127_T_R_1528595518 |
| rs2973043 | *GDNF* | 5 | rs2973043-127_T_R_1529565064 |
| rs884344 | *GDNF* | 5 | rs884344-127_T_F_1528586868 |
| rs12521946 | *GDNF* | 5 | rs12521946-127_B_R_1529564999 |
| rs2973042 | *GDNF* | 5 | rs2973042-127_T_F_1529565054 |
| rs2973041 | *GDNF* | 5 | rs2973041-127_T_F_1529565034 |
| rs10941370 | *GDNF* | 5 | rs10941370-127_T_R_1526511372 |
| rs3812047 | *GDNF* | 5 | rs3812047-127_B_R_1528585525 |
| rs2975100 | *GDNF* | 5 | rs2975100-127_B_F_1528585427 |
| rs1011814 | *FGF10* | 5 | rs1011814-127_T_F_1526340210 |
| rs11743802 | *FGF10* | 5 | rs11743802-127_B_F_1526340385 |
| rs2121875 | *FGF10* | 5 | rs2121875-127_T_R_1526340392 |
| rs11750845 | *FGF10* | 5 | rs11750845-127_B_F_1526340247 |
| rs1384449 | *FGF10* | 5 | rs1384449-127_T_F_1526340388 |
| rs10512849 | *FGF10* | 5 | rs10512849-127_B_F_1526340349 |
| rs984253 | *FOXC1* | 6 | rs984253-127_B_F_1503910605 |
| rs6968828 | *HOXA11* | 7 | rs6968828-127_T_R_1528626850 |
| rs17427875 | *HOXA11* | 7 | rs17427875-127_B_F_1529056509 |
| rs12673952 | *KIAA0241* | 7 | rs12673952-127_B_F_1500456181 |
| rs6959234 | *KIAA0241* | 7 | rs6959234-127_B_F_1529248465 |
| rs6967167 | *KIAA0241* | 7 | rs6967167-127_B_R_1529247185 |
| rs2290214 | *KIAA0241* | 7 | rs2290214-127_B_F_1529246297 |
| rs17365249 | *KIAA0241* | 7 | rs17365249-127_T_R_1529248246 |
| rs1993050 | *KIAA0241* | 7 | rs1993050-127_B_R_1529246221 |
| rs6462381 | *KIAA0241* | 7 | rs6462381-127_B_F_1529246227 |
| rs4431500 | *KIAA0241* | 7 | rs4431500-127_T_R_1500469742 |
| rs34778959 | *KIAA0241* | 7 | rs34778959-127_B_F_1529247449 |
| rs3801332 | *KIAA0241* | 7 | rs3801332-127_T_R_1524234030 |
| rs2110465 | *UPK3B* | 7 | rs2110465-127_T_R_1526361316 |
| rs4072400 | *EYA1* | 8 | rs4072400-127_T_F_1503035014 |
| rs10103397 | *EYA1* | 8 | rs10103397-127_B_R_1529565109 |
| rs9298164 | *EYA1* | 8 | rs9298164-127_B_F_1528642799 |
| rs3735935 | *EYA1* | 8 | rs3735935-127_T_F_1503243325 |
| rs4738118 | *EYA1* | 8 | rs4738118-127_B_R_1529565117 |
| rs1481800 | *EYA1* | 8 | rs1481800-127_T_F_1503270360 |
| rs4738119 | *EYA1* | 8 | rs4738119-127_T_F_1529565112 |
| rs16937518 | *EYA1* | 8 | rs16937518-127_B_R_1529565113 |
| rs13274732 | *EYA1* | 8 | rs13274732-127_T_F_1527363860 |
| rs13262002 | *EYA1* | 8 | rs13262002-127_T_R_1500333989 |
| rs17782527 | *EYA1* | 8 | rs17782527-127_T_R_1527363854 |
| rs1900078 | *EYA1* | 8 | rs1900078-127_B_R_1503937327 |
| rs1900079 | *EYA1* | 8 | rs1900079-127_B_F_1502573601 |
| rs7004007 | *EYA1* | 8 | rs7004007-127_T_R_1503377679 |
| rs2380716 | *EYA1* | 8 | rs2380716-127_B_F_1502514741 |
| rs1900081 | *EYA1* | 8 | rs1900081-127_T_R_1500095877 |
| rs10088075 | *EYA1* | 8 | rs10088075-127_T_R_1500096609 |
| rs7011671 | *EYA1* | 8 | rs7011671-127_T_R_1529565106 |
| rs733745 | *EYA1* | 8 | rs733745-127_B_R_1503275770 |
| rs6990158 | *EYA1* | 8 | rs6990158-127_B_F_1503248387 |
| rs8181006 | *EYA1* | 8 | rs8181006-127_T_F_1526516196 |
| rs10092844 | *EYA1* | 8 | rs10092844-127_T_R_1500095739 |
| rs11993876 | *EYA1* | 8 | rs11993876-127_B_F_1527363819 |
| rs1031178 | *EYA1* | 8 | rs1031178-127_B_R_1503239185 |
| rs10095184 | *EYA1* | 8 | rs10095184-127_T_R_1528644378 |
| rs1445410 | *EYA1* | 8 | rs1445410-127_B_R_1529565115 |
| rs7008755 | *EYA1* | 8 | rs7008755-127_B_R_1502518267 |
| rs6472582 | *EYA1* | 8 | rs6472582-127_T_R_1527363810 |
| rs12679412 | *EYA1* | 8 | rs12679412-127_B_F_1527363823 |
| rs12679427 | *EYA1* | 8 | rs12679427-127_T_R_1500096275 |
| rs10957546 | *EYA1* | 8 | rs10957546-127_T_R_1529565120 |
| rs4738128 | *EYA1* | 8 | rs4738128-127_B_F_1500096315 |
| rs17712160 | *EYA1* | 8 | rs17712160-127_B_R_1527363843 |
| rs17712165 | *EYA1* | 8 | rs17712165-127_T_F_1527363840 |
| rs3779748 | *EYA1* | 8 | rs3779748-127_T_R_1503363314 |
| rs1900077 | *EYA1* | 8 | rs1900077-127_B_R_1503370502 |
| rs4738129 | *EYA1* | 8 | rs4738129-127_B_R_1503292983 |
| rs6983443 | *EYA1* | 8 | rs6983443-127_T_R_1527363814 |
| rs6983593 | *EYA1* | 8 | rs6983593-127_B_F_1527363833 |
| rs2218488 | *EYA1* | 8 | rs2218488-127_T_R_1503241469 |
| rs6992926 | *EYA1* | 8 | rs6992926-127_B_R_1502497650 |
| rs1822917 | *EYA1* | 8 | rs1822917-127_T_R_1502583181 |
| rs11776476 | *EYA1* | 8 | rs11776476-127_T_R_1527363026 |
| rs2275806 | *GATA3* | 10 | rs2275806-127_T_F_1503252834 |
| rs1399180 | *GATA3* | 10 | rs1399180-127_T_R_1502589696 |
| rs3781093 | *GATA3* | 10 | rs3781093-127_T_F_1503910117 |
| rs3802604 | *GATA3* | 10 | rs3802604-127_B_F_1502546346 |
| rs3824662 | *GATA3* | 10 | rs3824662-127_T_R_1526099156 |
| rs570613 | *GATA3* | 10 | rs570613-127_T_F_1503373793 |
| rs569421 | *GATA3* | 10 | rs569421-127_B_F_1503910301 |
| rs528778 | *GATA3* | 10 | rs528778-127_B_F_1503910281 |
| rs263419 | *GATA3* | 10 | rs263419-127_B_R_1529564977 |
| rs878321 | *ITGA8* | 10 | rs878321-127_T_R_1502499796 |
| rs878319 | *ITGA8* | 10 | rs878319-127_T_R_1528660618 |
| rs11813586 | *ITGA8* | 10 | rs11813586-127_T_R_1529565240 |
| rs7910674 | *ITGA8* | 10 | rs7910674-127_B_R_1503962716 |
| rs1349534 | *ITGA8* | 10 | rs1349534-127_T_F_1502522682 |
| rs17137440 | *ITGA8* | 10 | rs17137440-127_T_F_1529565238 |
| rs11259736 | *ITGA8* | 10 | rs11259736-127_B_R_1529565135 |
| rs10752399 | *ITGA8* | 10 | rs10752399-127_T_F_1507483251 |
| rs2277204 | *ITGA8* | 10 | rs2277204-127_T_R_1503913433 |
| rs10906931 | *ITGA8* | 10 | rs10906931-127_T_R_1529565044 |
| rs1451668 | *ITGA8* | 10 | rs1451668-127_T_R_1529564992 |
| rs1057969 | *ITGA8* | 10 | rs1057969-127_B_F_1529565045 |
| rs980712 | *ITGA8* | 10 | rs980712-127_T_R_1503069219 |
| rs1319614 | *ITGA8* | 10 | rs1319614-127_T_F_1529565094 |
| rs1451667 | *ITGA8* | 10 | rs1451667-127_B_R_1529564989 |
| rs4747247 | *ITGA8* | 10 | rs4747247-127_B_R_1529565137 |
| rs2282384 | *ITGA8* | 10 | rs2282384-127_B_F_1529564995 |
| rs7083600 | *ITGA8* | 10 | rs7083600-127_B_F_1500145621 |
| rs7079006 | *ITGA8* | 10 | rs7079006-127_T_R_1500146042 |
| rs12414926 | *ITGA8* | 10 | rs12414926-127_B_F_1529565041 |
| rs1376690 | *ITGA8* | 10 | rs1376690-127_T_F_1503072104 |
| rs7918309 | *ITGA8* | 10 | rs7918309-127_B_R_1529565131 |
| rs12774861 | *ITGA8* | 10 | rs12774861-127_T_F_1529565176 |
| rs11253593 | *ITGA8* | 10 | rs11253593-127_T_F_1529565178 |
| rs2275617 | *ITGA8* | 10 | rs2275617-127_B_F_1502504680 |
| rs1341106 | *ITGA8* | 10 | rs1341106-127_B_F_1501190690 |
| rs6602051 | *ITGA8* | 10 | rs6602051-127_T_R_1529565134 |
| rs3765584 | *ITGA8* | 10 | rs3765584-127_B_R_1502545713 |
| rs1341099 | *ITGA8* | 10 | rs1341099-127_B_F_1500183385 |
| rs1891050 | *ITGA8* | 10 | rs1891050-127_T_F_1529565140 |
| rs11253615 | *ITGA8* | 10 | rs11253615-127_T_F_1529565142 |
| rs9333260 | *ITGA8* | 10 | rs9333260-127_T_R_1529565104 |
| rs2506011 | *RET* | 10 | RS2506011-127_T_R_1503381609 |
| rs2506021 | *RET* | 10 | rs2506021-127_T_R_1529564964 |
| rs12247456 | *RET* | 10 | rs12247456-127_B_R_1529565037 |
| rs2435347 | *RET* | 10 | rs2435347-127_T_F_1528665464 |
| rs2505535 | *RET* | 10 | rs2505535-127_T_R_1503050136 |
| rs1800858 | *RET* | 10 | rs1800858-127_B_R_1503909600 |
| rs3026737 | *RET* | 10 | rs3026737-127_B_F_1503949769 |
| rs2505515 | *RET* | 10 | rs2505515-127_B_F_1503913913 |
| rs1800860 | *RET* | 10 | rs1800860-127_B_R_1503909601 |
| rs741968 | *RET* | 10 | rs741968-127_B_R_1529565077 |
| rs2742234 | *RET* | 10 | rs2742234-127_T_R_1529565036 |
| rs1800861 | *RET* | 10 | rs1800861-127_B_R_1529564967 |
| rs1800863 | *RET* | 10 | rs1800863-127_T_R_1503956374 |
| rs715106 | *RET* | 10 | rs715106-127_B_R_1503255536 |
| rs2565201 | *RET* | 10 | rs2565201-127_T_F_1529565072 |
| rs17028 | *RET* | 10 | rs17028-127_T_R_1528665258 |
| rs4917908 | *PAX2* | 10 | rs4917908-127_B_F_1503085986 |
| rs4278455 | *PAX2* | 10 | rs4278455-127_T_F_1503959892 |
| rs6421335 | *PAX2* | 10 | rs6421335-127_T_R_1500465103 |
| rs4244341 | *PAX2* | 10 | rs4244341-127_T_R_1500138182 |
| rs11599767 | *PAX2* | 10 | rs11599767-127_B_F_1529565145 |
| rs10786607 | *PAX2* | 10 | rs10786607-127_T_F_1507483487 |
| rs11190695 | *PAX2* | 10 | rs11190695-127_B_F_1500493227 |
| rs2863046 | *PAX2* | 10 | rs2863046-127_B_R_1503054136 |
| rs10883543 | *PAX2* | 10 | rs10883543-127_B_F_1528663499 |
| rs2077642 | *PAX2* | 10 | rs2077642-127_B_F_1528665165 |
| rs1800898 | *PAX2* | 10 | rs1800898-127_B_R_1500137251 |
| rs996359 | *PAX2* | 10 | rs996359-127_T_F_1503030975 |
| rs11816136 | *PAX2* | 10 | rs11816136-127_T_F_1500493858 |
| rs1061413 | *GFRA1* | 10 | rs1061413-127_T_R_1502571337 |
| rs11598215 | *GFRA1* | 10 | rs11598215-127_T_F_1529565010 |
| rs3781504 | *GFRA1* | 10 | rs3781504-127_B_R_1503959032 |
| rs10490904 | *GFRA1* | 10 | rs10490904-127_B_R_1528663543 |
| rs7087152 | *GFRA1* | 10 | rs7087152-127_T_R_1529565156 |
| rs7087213 | *GFRA1* | 10 | rs7087213-127_B_R_1529565029 |
| rs11197523 | *GFRA1* | 10 | rs11197523-127_B_F_1529565003 |
| rs3901216 | *GFRA1* | 10 | rs3901216-127_B_F_1526509357 |
| rs7070180 | *GFRA1* | 10 | rs7070180-127_B_F_1529565025 |
| rs3781517 | *GFRA1* | 10 | rs3781517-127_B_R_1529565151 |
| rs3847476 | *GFRA1* | 10 | rs3847476-127_B_F_1529565031 |
| rs2694798 | *GFRA1* | 10 | rs2694798-127_B_F_1502575164 |
| rs3843600 | *GFRA1* | 10 | rs3843600-127_T_R_1529565024 |
| rs3781523 | *GFRA1* | 10 | rs3781523-127_B_R_1503352656 |
| rs3781531 | *GFRA1* | 10 | rs3781531-127_B_R_1503025999 |
| rs3781532 | *GFRA1* | 10 | rs3781532-127_B_R_1503243867 |
| rs10885864 | *GFRA1* | 10 | rs10885864-127_B_R_1503288801 |
| rs2420242 | *GFRA1* | 10 | rs2420242-127_B_F_1528665329 |
| rs10749189 | *GFRA1* | 10 | rs10749189-127_T_R_1502551243 |
| rs10885868 | *GFRA1* | 10 | rs10885868-127_B_F_1529565011 |
| rs4751955 | *GFRA1* | 10 | rs4751955-127_B_R_1529565017 |
| rs11197567 | *GFRA1* | 10 | rs11197567-127_T_F_1528663522 |
| rs7901076 | *GFRA1* | 10 | rs7901076-127_B_R_1529565015 |
| rs11197571 | *GFRA1* | 10 | rs11197571-127_T_F_1529565154 |
| rs7904143 | *GFRA1* | 10 | rs7904143-127_B_F_1529565021 |
| rs10787635 | *GFRA1* | 10 | rs10787635-127_T_R_1500492485 |
| rs11197576 | *GFRA1* | 10 | rs11197576-127_T_R_1529565008 |
| rs3781550 | *GFRA1* | 10 | rs3781550-127_T_F_1503925506 |
| rs1107030 | *GFRA1* | 10 | rs1107030-127_B_F_1526513243 |
| rs881726 | *GFRA1* | 10 | rs881726-127_B_R_1503099342 |
| rs10787637 | *GFRA1* | 10 | rs10787637-127_T_F_1500492486 |
| rs7907314 | *GFRA1* | 10 | rs7907314-127_T_F_1529565020 |
| rs3781556 | *GFRA1* | 10 | rs3781556-127_T_R_1503293619 |
| rs730357 | *GFRA1* | 10 | rs730357-127_B_F_1503246500 |
| rs7920266 | *GFRA1* | 10 | rs7920266-127_B_R_1529565013 |
| rs385209 | *EMX2* | 10 | rs385209-127_B_R_1500184774 |
| rs2240776 | *EMX2* | 10 | rs2240776-127_T_R_1503301180 |
| rs5030335 | *WT1* | 11 | rs5030335-127_B_F_1528672271 |
| rs5030320 | *WT1* | 11 | rs5030320-127_T_F_1527354882 |
| rs16754 | *WT1* | 11 | rs16754-127_T_F_1529565100 |
| rs1569776 | *WT1* | 11 | rs1569776-127_B_F_1528674139 |
| rs10767935 | *WT1* | 11 | rs10767935-127_B_F_1528668337 |
| rs3901671 | *WT1* | 11 | rs3901671-127_T_R_1528668426 |
| rs3858444 | *WT1* | 11 | rs3858444-127_T_F_1527354988 |
| rs3818055 | *WT1* | 11 | rs3818055-127_T_F_1528672066 |
| rs2900741 | *WT1* | 11 | rs2900741-127_T_F_1527354886 |
| rs7936152 | *WT1* | 11 | rs7936152-127_T_F_1527355110 |
| rs3930513 | *WT1* | 11 | rs3930513-127_T_R_1527355122 |
| rs5030141 | *WT1* | 11 | rs5030141-127_T_F_1529564998 |
| rs1799925 | *WT1* | 11 | rs1799925-127_T_R_1529564948 |
| rs520654 | *IGHMBP2* | 11 | rs520654-127_B_F_1529245933 |
| rs1249463 | *IGHMBP2* | 11 | rs1249463-127_T_F_1529245900 |
| rs560096 | *IGHMBP2* | 11 | rs560096-127_B_F_1528489439 |
| rs10896379 | *IGHMBP2* | 11 | rs10896379-127_T_R_1529245726 |
| rs10896380 | *IGHMBP2* | 11 | rs10896380-127_T_F_1528666842 |
| rs10896381 | *IGHMBP2* | 11 | rs10896381-127_T_F_1529245786 |
| rs637120 | *IGHMBP2* | 11 | rs637120-127_T_R_1529245798 |
| rs4930627 | *IGHMBP2* | 11 | rs4930627-127_B_F_1529245859 |
| rs674654 | *IGHMBP2* | 11 | rs674654-127_B_R_1529245847 |
| rs653264 | *IGHMBP2* | 11 | rs653264-127_B_F_1529245767 |
| rs2282504 | *IGHMBP2* | 11 | rs2282504-127_B_R_1529245527 |
| rs11228413 | *IGHMBP2* | 11 | rs11228413-127_B_F_1529245971 |
| rs598255 | *IGHMBP2* | 11 | rs598255-127_T_R_1503037698 |
| rs622082 | *IGHMBP2* | 11 | rs622082-127_T_F_1528666788 |
| rs2236654 | *IGHMBP2* | 11 | rs2236654-127_T_F_1529245502 |
| rs546382 | *IGHMBP2* | 11 | rs546382-127_B_F_1529245803 |
| rs17612126 | *IGHMBP2* | 11 | rs17612126-127_B_R_1529245855 |
| rs598143 | *WNT11* | 11 | rs598143-127_T_F_1526162950 |
| rs12277860 | *WNT11* | 11 | rs12277860-127_T_R_1526161280 |
| rs17749202 | *WNT11* | 11 | rs17749202-127_B_F_1524942819 |
| rs10899175 | *WNT11* | 11 | rs10899175-127_T_R_1524942596 |
| rs882151 | *WNT11* | 11 | rs882151-127_B_F_1526094579 |
| rs3781730 | *WNT11* | 11 | rs3781730-127_T_R_1526155690 |
| rs4944092 | *WNT11* | 11 | rs4944092-127_T_F_1526150666 |
| rs689095 | *WNT11* | 11 | rs689095-127_T_F_1524942702 |
| rs1790191 | *UPK2* | 11 | rs1790191-127_B_R_1525050605 |
| rs623500 | *UPK2* | 11 | rs623500-127_T_F_1525130326 |
| rs5443 | *GNB3* | 12 | rs5443-127_B_F_1524468811 |
| rs1554753 | *RARG* | 12 | rs1554753-127_T_F_1525908398 |
| rs6580936 | *RARG* | 12 | rs6580936-127_B_R_1526093683 |
| rs4512901 | *HOXC11* | 12 | rs4512901-127_T_R_1529564970 |
| rs12427129 | *HOXC11* | 12 | rs12427129-127_B_F_1529564957 |
| rs11911 | *SPRY2* | 13 | rs11911-127_T_R_1500169961 |
| rs504122 | *SPRY2* | 13 | rs504122-127_B_F_1500040694 |
| rs17563 | *BMP4* | 14 | rs17563-127_B_F_1501192486 |
| rs2071047 | *BMP4* | 14 | rs2071047-127_T_R_1529564966 |
| rs762642 | *BMP4* | 14 | rs762642-127_B_F_1526093633 |
| rs2761887 | *BMP4* | 14 | rs2761887-127_B_R_1529564971 |
| rs1528734 | *GREM1* | 15 | rs1528734-127_B_R_1526338599 |
| rs7497354 | *GREM1* | 15 | rs7497354-127_T_F_1526340304 |
| rs3743105 | *GREM1* | 15 | rs3743105-127_B_R_1526340293 |
| rs17228641 | *GREM1* | 15 | rs17228641-127_B_R_1526339355 |
| rs10318 | *GREM1* | 15 | rs10318-127_T_R_1526340298 |
| rs7176378 | *GREM1* | 15 | rs7176378-127_B_R_1526338601 |
| rs11070692 | *FGF7* | 15 | rs11070692-127_T_R_1526186610 |
| rs16962440 | *FGF7* | 15 | rs16962440-127_T_R_1526186646 |
| rs10519225 | *FGF7* | 15 | rs10519225-127_B_R_1526183483 |
| rs4338740 | *FGF7* | 15 | rs4338740-127_T_R_1526183074 |
| rs11070693 | *FGF7* | 15 | rs11070693-127_T_F_1526186616 |
| rs17400706 | *FGF7* | 15 | rs17400706-127_B_R_1526186651 |
| rs11634375 | *FGF7* | 15 | rs11634375-127_B_F_1526183975 |
| rs4480740 | *FGF7* | 15 | rs4480740-127_B_R_1526183079 |
| rs4407014 | *FGF7* | 15 | rs4407014-127_T_F_1526186674 |
| rs2413958 | *FGF7* | 15 | rs2413958-127_B_F_1526088627 |
| rs9972329 | *FGF7* | 15 | rs9972329-127_T_R_1526183460 |
| rs751979 | *SALL1* | 16 | rs751979-127_T_F_1527981936 |
| rs11645288 | *SALL1* | 16 | rs11645288-127_B_R_1527959235 |
| rs1965024 | *SALL1* | 16 | rs1965024-127_B_F_1503024104 |
| rs1015438 | *SALL1* | 16 | rs1015438-127_B_F_1527969097 |
| rs7502966 | *THRA* | 17 | rs7502966-127_T_R_1524465306 |
| rs1568400 | *THRA* | 17 | rs1568400-127_T_F_1524465312 |
| rs939348 | *THRA* | 17 | rs939348-127_B_F_1524465307 |
| rs2715553 | *RARA* | 17 | rs2715553-127_T_R_1526099954 |
| rs9303285 | *RARA* | 17 | rs9303285-127_B_F_1527355019 |
| rs482284 | *RARA* | 17 | rs482284-127_T_F_1526094040 |
| rs8074816 | *WNT9B* | 17 | rs8074816-127_T_R_1526340330 |
| rs2165846 | *WNT9B* | 17 | rs2165846-127_T_F_1526340280 |
| rs6504591 | *WNT9B* | 17 | rs6504591-127_B_F_1526340271 |
| rs4968281 | *WNT9B* | 17 | rs4968281-127_B_F_1526093543 |
| rs1530364 | *WNT9B* | 17 | rs1530364-127_B_R_1526340269 |
| rs2267584 | *UPK1A* | 19 | rs2267584-127_B_R_1526360963 |
| rs10413852 | *UPK1A* | 19 | rs10413852-127_B_R_1526361243 |
| rs2285421 | *UPK1A* | 19 | rs2285421-127_T_R_1526360972 |
| rs11882996 | *UPK1A* | 19 | rs11882996-127_T_F_1526361218 |
| rs1982073 | *TGFB1* | 19 | rs1982073-127_B_F_1524232927 |
| rs1800469 | *TGFB1* | 19 | rs1800469-127_T_R_1503909587 |
| rs1800468 | *TGFB1* | 19 | rs1800468-127_B_R_1524837341 |
| rs5995802 | *TNRC6B* | 22 | rs5995802-127_T_F_1500073127 |
| rs7288667 | *TNRC6B* | 22 | rs7288667-127_T_F_1502498587 |
| rs7292838 | *TNRC6B* | 22 | rs7292838-127_T_F_1529246316 |
| rs9611265 | *TNRC6B* | 22 | rs9611265-127_B_R_1500325832 |
| rs9611266 | *TNRC6B* | 22 | rs9611266-127_B_F_1529246969 |
| rs12628757 | *TNRC6B* | 22 | rs12628757-127_B_F_1500337271 |
| rs2413611 | *TNRC6B* | 22 | rs2413611-127_B_F_1503271983 |
| rs2143177 | *TNRC6B* | 22 | rs2143177-127_T_R_1529246808 |
| rs11089974 | *TNRC6B* | 22 | rs11089974-127_B_F_1500072369 |
| rs9611280 | *TNRC6B* | 22 | rs9611280-127_T_F_1528769608 |
| rs12628783 | *TNRC6B* | 22 | rs12628783-127_T_F_1529246754 |
| rs138027 | *TNRC6B* | 22 | rs138027-127_B_R_1503329426 |
| rs12628042 | *TNRC6B* | 22 | rs12628042-127_T_R_1529246552 |
| rs9611302 | *TNRC6B* | 22 | rs9611302-127_T_F_1500325833 |
| rs763071 | *TNRC6B* | 22 | rs763071-127_B_R_1502590645 |
| rs713898 | *TNRC6B* | 22 | rs713898-127_T_F_1502558372 |
| rs139914 | *TNRC6B* | 22 | rs139914-127_B_R_1502562293 |
| rs1057353 | *UPK3A* | 22 | rs1057353-127_T_F_1503031461 |
| rs1135360 | *UPK3A* | 22 | rs1135360-127_B_R_1526360985 |
| rs3788643 | *UPK3A* | 22 | rs3788643-127_T_F_1502575701 |
| rs1057356 | *UPK3A* | 22 | rs1057356-127_B_R_1503288866 |
