# Supplementary material for: Genes in the Ureteric Budding Pathway: Association Study on Vesico-Ureteral Reflux Patients
Source: PLoS One. 2012 Apr 27;7(4):e31327. doi: 10.1371/journal.pone.0031327 (PMC3338743; doi:10.1371/journal.pone.0031327)
Supplement: Table S3 — Three inherited UPK3A mutations identified in the duplex collecting system subgroup. Results of in silico analysis, online database queries, and renal ultrasound in parents. (DOCX) [file pone.0031327.s005.docx]

**Table S3.** Three inherited *UPK3A* mutations identified in the duplex collecting system subgroup. Results of in silico analysis, online database queries, and renal ultrasound in parents.

| Duplex collecting system and VUR case ID | location | position | amino acid | mutation (cDNA) | mutation (protein) | Sanger sequencing result in 96 control chromosomes | HGMD | ENTREZ SNP | domain | Grantham difference | align GVGD result (Grantham variation, Grantham Deviation) | previously published | renal ultrasound in parents |
| --- | --- | --- | --- | --- | --- | --- | --- | --- | --- | --- | --- | --- | --- |
| 81079 | exon 3 | 211 | 71 | c.211A>G | p.Ile71Val | absent | not reported | not reported | luminal | 29 | ClassC0*,  179,47 | | no urinary tract abnormalities detected |
| 27658 | exon 6 | 811 | 271 | c.811C>T | p.Arg271Trp | absent | not reported | not reported | cytoplasmic | 101 | Class C0*,  243,26 | | Parent that carries *UPK3A* mutation: central complex interrupted by a parenchymal ridge, suspect for a duplex collecting system. Other parent: no relevant abnormalities. |
| 81152 | exon 6 | 818 | 273 | c.818C>T | p.Pro273Leu | absent | reported: accession# CM056713 | not reported | cytoplasmic | 98 | Class C0*, 208,63 | Jenkins et al. 2006: "probably behaves like wildtype" (Jenkins (2005) J Am Soc Nephrol 16, 2141) | no consent for renal ultrasound |

* align GVGD class C0 means "probably neutral variant”
