# Supplementary material for: Genes in the Ureteric Budding Pathway: Association Study on Vesico-Ureteral Reflux Patients
Source: PLoS One. 2012 Apr 27;7(4):e31327. doi: 10.1371/journal.pone.0031327 (PMC3338743; doi:10.1371/journal.pone.0031327)
Supplement: Table S4 — Tagging and functional SNPs in this association study that passed our quality control criteria. (DOCX) [file pone.0031327.s006.docx]

**Table S4.** Tagging and functional SNPs in this association study that passed our quality control criteria.

| gene | percentage designed tag SNPs passed QC | total nr of designed tag SNPs | percentage of functional SNPs passed QC | total nr of designed functional SNPs |
| --- | --- | --- | --- | --- |
| *HOXD11* | 100 | 2 | n/a | 0 |
| *BMP4* | 100 | 4 | n/a | 0 |
| *FSTL1* | 100 | 13 | n/a | 0 |
| *UPK3A* | 100 | 3 | 50 | 2 |
| *HOXC11* | 100 | 1 | 25 | 4 |
| *SALL1* | 100 | 3 | 20 | 5 |
| *FOXC1* | 100 | 1 | 0 | 2 |
| *UPK3B* | 100 | 1 | 0 | 4 |
| *CTNNB1* | 100 | 2 | 0 | 10 |
| *EMX2* | 100 | 2 | 0 | 2 |
| *SIX2* | 100 | 2 | 0 | 1 |
| *FGF10* | 100 | 6 | 0 | 1 |
| *GREM1* | 100 | 6 | 0 | 2 |
| *ITGA8* | 94 | 34 | 0 | 1 |
| *PAX2* | 93 | 14 | n/a | 0 |
| *ROBO2* | 93 | 81 | 0 | 6 |
| *FGF7* | 92 | 12 | 0 | 2 |
| *GDNF* | 92 | 13 | 0 | 2 |
| *RARB* | 91 | 105 | n/a | 0 |
| *IGHMBP2* | 90 | 10 | 47 | 17 |
| *GATA3* | 90 | 10 | 0 | 3 |
| *SLIT2* | 90 | 61 | 0 | 1 |
| *WNT11* | 89 | 9 | n/a | 0 |
| *UPK1B* | 89 | 19 | 0 | 1 |
| *EYA1* | 88 | 48 | 33 | 3 |
| *GFRA1* | 85 | 41 | 0 | 3 |
| *WNT9B* | 83 | 6 | n/a | 0 |
| *KIAA0241* | 80 | 10 | 67 | 3 |
| *WT1* | 80 | 15 | 25 | 4 |
| *TNRC6B* | 80 | 20 | 17 | 6 |
| *RET* | 78 | 18 | 15 | 13 |
| *SPRY1* | 67 | 3 | 0 | 1 |
| *SPRY2* | 67 | 3 | 0 | 2 |
| *UPK1A* | 67 | 6 | 0 | 3 |
| *OSR1* | 60 | 5 | 0 | 2 |
| *RARA* | 60 | 5 | 0 | 2 |
| *THRA* | 60 | 5 | 0 | 2 |
| *ZIC1* | 50 | 2 | n/a | 0 |
| *HOXA11* | 50 | 4 | n/a | 0 |
| *RARG* | 50 | 4 | 0 | 1 |
| *NPNT* | 45 | 11 | 25 | 4 |
| *UPK2* | 33 | 3 | 50 | 2 |
| *GNB3** | n/a | 0 | 100 | 1 |
| *TGFB1** | n/a | 0 | 100 | 3 |
| *AGTR2* | n/a | 0 | 0 | 4 |
| *E2F4* | n/a | 0 | 0 | 2 |
| *FOXC2* | n/a | 0 | 0 | 2 |
| *GDF11* | n/a | 0 | 0 | 1 |
| *LHX1* | n/a | 0 | 0 | 2 |
| *MXRA8* | n/a | 0 | 0 | 1 |
| *SIX1* | n/a | 0 | 0 | 1 |
| *VEGFA** | n/a | 0 | 0 | 1 |

* Genes were not tagged, only specific SNPs, previously found to be associated with VUR, were included for replication.
