# Supplementary material for: Genes in the Ureteric Budding Pathway: Association Study on Vesico-Ureteral Reflux Patients
Source: PLoS One. 2012 Apr 27;7(4):e31327. doi: 10.1371/journal.pone.0031327 (PMC3338743; doi:10.1371/journal.pone.0031327)
Supplement: Table S5 — TaqMan assay IDs for SNPs genotyped in stage two (Applied Biosystems). (DOCX) [file pone.0031327.s007.docx]

**Table S5.** TaqMan assay IDs for SNPs genotyped in stage two (Applied Biosystems).

| SNP | gene | assay ID |
| --- | --- | --- |
| rs1481800 | *EYA1* | C___7690584_10 |
| rs3735935 | *EYA1* | C__25804074_10 |
| rs11197571 | *GFRA1* | C__31975369_10 |
| rs7497354 | *GREM1* | C____466953_10 |
| rs6780105 | *RARB* | C__28985572_10 |
| rs755661 | *RARB* | C___1957990_20 |
| rs1666130 | *ROBO2* | C___8241262_10 |
| rs1721175 | *ROBO2* | C___2165875_10 |
| rs4476545 | *ROBO2* | C__30166401_20 |
| rs1057353 | *UPK3A* | C__11879932_20 |
| rs1135360 | *UPK3A* | C__11483267_1 |
| rs3788643 | *UPK3A* | C__27485900_10 |
