# Supplementary material for: Genes in the Ureteric Budding Pathway: Association Study on Vesico-Ureteral Reflux Patients
Source: PLoS One. 2012 Apr 27;7(4):e31327. doi: 10.1371/journal.pone.0031327 (PMC3338743; doi:10.1371/journal.pone.0031327)
Supplement: Table S6 — Primer sequences used for UPK3A sequencing and sequencing of rs1057353 (indicated with *). (DOCX) [file pone.0031327.s008.docx]

**Table S6.** Primer sequences used for *UPK3A* sequencing and sequencing of rs1057353 (indicated with *).

| Exon_nr_strand | Sequence |
| --- | --- |
| exon_1_forw | ACACAGTAGGCGCTTTGAT |
| exon_1_rev | CGTAAACGTTGGCTATCACT |
| exon_2_forw | ATTTCTGAGCAGGATGACTG |
| exon_2_rev | TCCCTCACTAACTGGATGTC |
| exon_3_1_forw | CTGAGAGGGCAGAGACTAAG |
| exon_3_1_rev | TTTACACCCACCTGTACTCC |
| exon_3_2_forw* | GGCATTTGATGAATAACTGAG |
| exon_3_2_rev* | GCCTCTTCTGAACTTGAGG |
| exon_4_forw | CAGTAGCCGTCTACATTTCC |
| exon_4_rev | CCTGGCTACTTTTGTTTTTG |
| exon_5_forw | AAGTTGGAAAGTGGAATGTG |
| exon_5_rev | TGAGCAACTTGACTTTGATG |
| exon_6_1_forw | GTGGACCTCTTCCTTATTCC |
| exon_6_1_rev | TTTCACCTTCCCTGAAGTC |
| exon_6_2_forw | ATCACTCAGGAGGCTGTTC |
| exon_6_2_rev | CACGATCATAGCTCATTGC |
